# Phenotypic variability in LQT3 human induced pluripotent stem cell-derived cardiomyocytes and their response to antiarrhythmic pharmacologic therapy: An *in silico* approach

**DOI:** 10.1016/j.hrthm.2017.07.026

**Published:** 2017-11

**Authors:** Michelangelo Paci, Elisa Passini, Stefano Severi, Jari Hyttinen, Blanca Rodriguez

**Affiliations:** ∗ BioMediTech Institute and Faculty of Biomedical Sciences and Engineering, Tampere University of Technology, Tampere, Finland; † Department of Computer Science, University of Oxford, Oxford, United Kingdom; ‡ Department of Electrical, Electronic and Information Engineering “Guglielmo Marconi”, University of Bologna, Cesena (FC), Italy

**Keywords:** Action potential, Drug test, Human induced pluripotent stem cell-derived cardiomyocyte, *In silico* modeling, Long QT syndrome type 3, Population of models, AP, action potential, APA, action potential amplitude, APD, action potential duration, hiPSC-CM, human induced pluripotent stem cell-derived cardiomyocyte, I_CaL_, L-type calcium current, I_K1_, inward rectifying potassium current, I_Kr_, rapid delayed rectifying potassium current, I_Na_, fast sodium current, I_NaL_, late sodium current, I_pCa_, calcium sarcolemmal pump, LQT3, long QT syndrome type 3, MDP, maximum diastolic potential, Peak, peak potential, rate, rate of spontaneous action potentials, V_Max_, maximum upstroke velocity

## Abstract

**Background:**

Human induced pluripotent stem cell-derived cardiomyocytes (hiPSC-CMs) are *in vitro* models with the clear advantages of their human origin and suitability for human disease investigations. However, limitations include their incomplete characterization and variability reported in different cell lines and laboratories.

**Objective:**

The purpose of this study was to investigate *in silico* ionic mechanisms potentially explaining the phenotypic variability of hiPSC-CMs in long QT syndrome type 3 (LQT3) and their response to antiarrhythmic drugs.

**Methods:**

Populations of *in silico* hiPSC-CM models were constructed and calibrated for control (n = 1,463 models) and LQT3 caused by I_NaL_ channelopathy (n = 1,401 models), using experimental recordings for late sodium current (I_NaL_) and action potentials (APs). Antiarrhythmic drug therapy was evaluated by simulating mexiletine and ranolazine multichannel effects.

**Results:**

As in experiments, LQT3 hiPSC-CMs yield prolonged action potential duration at 90% repolarization (APD_90_) (+34.3% than controls) and large electrophysiological variability. LQT3 hiPSC-CMs with symptomatic APs showed overexpression of I_CaL_, I_K1_, and I_NaL_, underexpression of I_Kr_, and increased sensitivity to both drugs compared to asymptomatic LQT3 models. Simulations showed that both mexiletine and ranolazine corrected APD prolongation in the LQT3 population but also highlighted differences in drug response. Mexiletine stops spontaneous APs in more LQT3 hiPSC-CMs models than ranolazine (784/1,401 vs 53/1,401) due to its stronger action on I_Na_.

**Conclusion:**

*In silico* simulations demonstrate our ability to recapitulate variability in LQT3 and control hiPSC-CM phenotypes, and the ability of mexiletine and ranolazine to reduce APD prolongation, in agreement with experiments. The *in silico* models also identify potential ionic mechanisms of phenotypic variability in LQT3 hiPSC-CMs, explaining APD prolongation in symptomatic vs asymptomatic LQT3 hiPSC-CMs.

## Introduction

The development of disease-specific human induced pluripotent stem cell-derived cardiomyocytes (hiPSC-CMs) offers promising alternatives to current *in vitro* and animal methods, particularly for the development of new treatments and the assessment of existing drugs for specific patient groups.^1^, ^2^, ^3^, ^4^ However, interpretation of experiments is hampered by the high variability of hiPSC-CMs datasets, which could be attributed to many factors, including (1) substantial differences among patients (eg, control cells in Fatima et al^2^ vs Lahti et al^5^); (2) immature phenotypes of hiPSC-CMs differentiated using *in vitro* techniques^6^; and (3) varying culturing conditions used in different laboratories. Little is known about the ionic mechanisms underlying variability in hiPSC-CMs phenotypes and their response to pharmacologic action.

Long QT syndrome type 3 (LQT3) is the third most common form of long QT syndrome, caused by mutations in the SCN5A gene, which encodes for the Na^+^ channels. At the cellular level, the LQT3 characteristic mechanism is the gain of function of the Na^+^ channels, which transport fast and late Na^+^ currents (I_Na_ and I_NaL_, respectively). Such gain of function causes an Na^+^ inward leak during the action potential (AP), which prolongs its repolarization. Few therapies are currently available,^7^ which in general are based on drugs blocking the Na^+^ currents, with particular effect on I_NaL_, such as mexiletine^8^ and ranolazine.^9^ Beta-blockers, which proved to be effective on LQT1 and LQT2, are less effective on LQT3 and can lead to bradycardia.

This study aimed to investigate key factors determining variability of LQT3 hiPSC-CM phenotypes and their response to antiarrhythmic drugs, using populations of *in silico* hiPSC-CMs calibrated with experimental recordings for control and LQT3 hiPSC-CMs. Given its experimental characterization, we focus on the inherited form of LQT3 induced by the V1763M mutation,^1^ which causes enlarged I_NaL_ and consequently prolonged AP. We also evaluated the potential antiarrhythmic effects of mexiletine and ranolazine, two antiarrhythmic drugs with multichannel action and suggested efficacy in LQT3 treatment.^7^, ^10^ Our approach provides an investigative platform toward precision medicine by extending *in vitro* studies to enable unraveling of the likely ionic mechanisms underlying variability in hiPSC-CM phenotypes for specific mutations and to evaluate their response to specific antiarrhythmic therapy.

## Methods

### Control and LQT3 hiPSC-CM models

The Paci2015 hiPSC-CM AP model was modified to include the I_NaL_ formulation for control and V1763M I_NaL_ mutation as explained in the Supplementary Material (Sections S1.1, S1.2, and Table S1).^11^, ^12^ To investigate hiPSC-CM phenotypical variability under control conditions, a random population of hiPSC-CM control models was developed as proposed in Britton et al,^13^ calibrated using experimental data from Ma et al,^14^ Moretti et al,^15^ Ma et al,^1^ Fatima et al,^2^ Lahti et al,^5^ and Kujala et al^16^ as further explained in the Supplementary Material (Sections S1.3 and S1.4, and Supplementary Table S2). The LQT3 mutant population of hiPSC-CM models was then developed by incorporating the V1763M mutation I_NaL_ formulation in all models included in the control population (ie, no further calibration was performed on the mutant population, following an approach similar to that of Passini et al^17^). Models in the mutant population were classified as asymptomatic and symptomatic as explained in Section S1.5 of the Supplementary Material.

### *In silico* drug tests

Effects of mexiletine and ranolazine at 5, 10, and 20 μM doses were assessed *in silico* on the control and mutant populations considering their multichannel effects on I_Na_, I_NaL_, the rapid delayed rectifying potassium current (I_Kr_), and the L-type calcium current (I_CaL_) using the single pore block model, consistent with data from ion channel assays (see Supplementary Material, Section S1.6, and Supplementary Table S3). Examples of drug effects on the Na^+^ current are reported in Supplementary Figures S1 and S2. To compare the effect of drug action on hiPSC vs adult cardiomyocytes, simulations were also conducted considering the same drug doses on 10 illustrative control and mutant models of human adult ventricular cardiomyocytes, based on the O’Hara-Rudy model^18^ (see Supplementary Material, Section S1.6). Unless otherwise specified, results are reported as mean ± SD.

## Results

### LQT3 mutation

**Figure 1:**
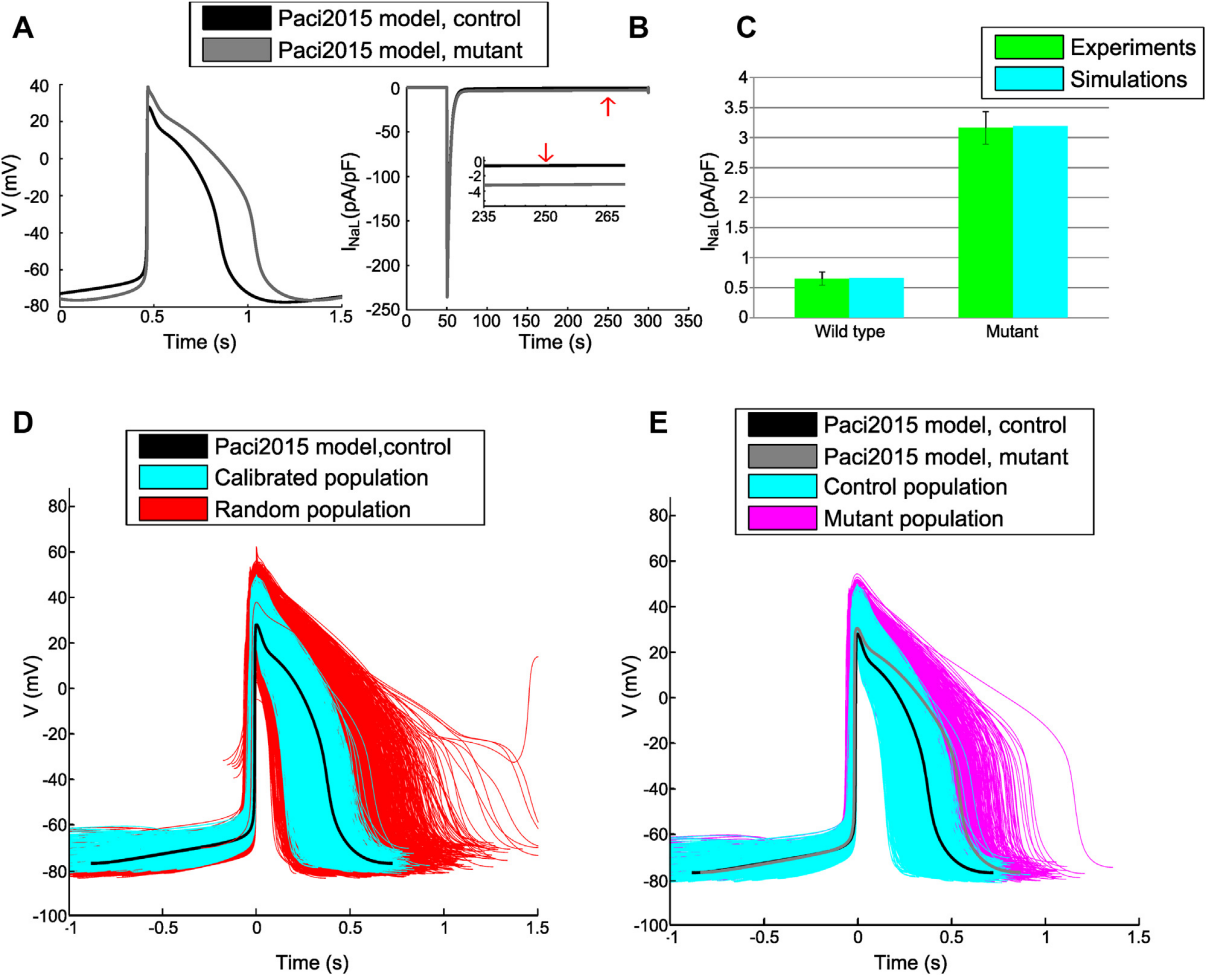
**A:** Comparison between Paci2015 hiPSC-CM model in control and LQT3 conditions. **B:** Simulated control and mutant Na^+^ currents (I_Na_ + I_NaL_). *Inset:* persistent I_NaL_ 200 ms after stimulus, for comparison with experimental data. *Red arrows* indicate simulated I_NaL_ values used for comparison with experimental data. **C:** Comparison between simulated and experimental^1^ persistent I_NaL_ The mutant I_NaL_ is 4.86 times larger than control current. **D:** APs generated by maximum conductance sampling. *Red* indicates rejected models; *cyan* indicates models included in the control population. Baseline AP from Paci2015 is represented in *black.***E:** Comparison between control *(cyan)* and mutant *(magenta)* populations. The mutant population features prolonged APs. The baseline Paci2015 model is represented in *black;* its mutant version in shown in *gray.* AP = action potential; hiPSC-CM = human induced pluripotent stem cell-derived cardiomyocyte; LQT3 = long QT syndrome type 3.

The hiPSC-CMs APs and the simulated I_NaL_ of the baseline models for the control and mutant conditions are described in Figures 1A and 1B, respectively, and in Supplementary Table S4. Figure 1C shows the excellent match of simulations with experimental data by Ma et al,^1^ reproducing the mean AP prolongation of the LQT3 V1763M mutation: +43% in simulation vs +48% in the experiments (control 434 ± 108 ms vs mutant 645 ± 239 ms).

### Control and LQT3 hiPSC-CMs populations

**Figure 2:**
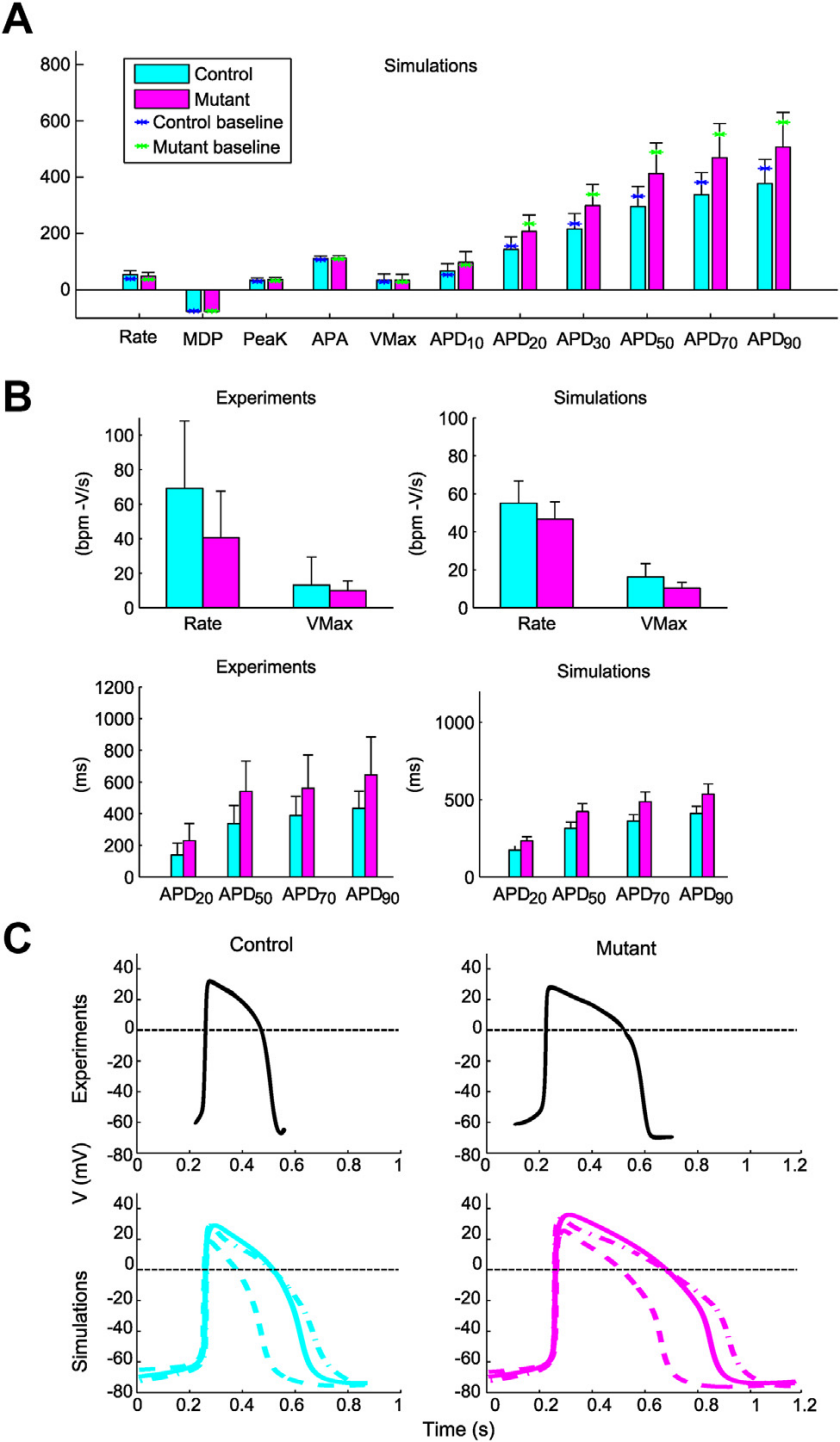
**A:** Simulated AP biomarkers in control vs mutant hiPSC-CM populations, showing APD prolongation with the mutation. **B:** Experimental and simulated AP biomarkers in control and LQT3 hiPSC-CM. A subpopulation of 410 hiPSC-CM models was extracted from the control population by calibration only with the dataset of Ma et al.^1^ The 150 models in agreement with the experimental data reported by Ma et al^1^ were extracted from the mutant population. **C:** APs from control and mutant subpopulations for experiments (redrawn from Ma et al^1^ with permission from Elsevier) and six illustrative *in silico* APs (three control and three mutant). The simulated *solid line* APs represent baseline control and mutant models; *dashed* and *dashed-dotted* line APs represent two additional models from the populations in control and mutant conditions, respectively. The top left and top right panels of Figure 2C were adapted from Figure 4 from International Journal of Cardiology, Volume 168, Issue 6, Ma D, Wei H, Zhao Y, Lu J, Li G, Sahib NB, Tan TH, Wong KY, Shim W, Wong P, Cook SA, Liew R, Modeling type 3 long QT syndrome with cardiomyocytes derived from patient-specific induced pluripotent stem cells, Pages 5277-5286, Copyright (2013), with permission from Elsevier. Anyone wishing to reuse either the original or the adapted figure must require formal permission from Elsevier to do so. APA = AP amplitude; APD = AP duration; MDP = maximum diastolic potential; Peak = peak voltage; Rate = rate of spontaneous APs; V_Max_ = maximum upstroke velocity. Other abbreviations as in Figure 1.

Figure 1D shows the APs of the random population (n = 10,000) and of the calibrated control population (n = 1,463). Figure 1E compares the APs of the control and mutant populations (n = 1,401). Figure 2A provides a quantitative description of the APs biomarkers obtained with both populations, clearly showing action potential duration (APD) prolongation (eg, ΔAPD_90_ = +34.3%) in the mutant population. Supplementary Figure S3 shows how biomarkers computed from the control population cover the experimental biomarker space.

Because the LQT3 V1763M mutation has been experimentally characterized in only one hiPSC-CM dataset,^1^ we also obtained a subpopulation of mutant models calibrated using only this dataset to assess the capability of the simulated population in reproducing the mutation experimental effects. Figure 2B shows the agreement in the effect of the mutation in simulations vs experimental data or all the biomarkers (top panels: rate of spontaneous APs (rate) and maximum upstroke velocity (V_Max_); bottom panels: APD). Finally, Figure 2C shows a comparison between illustrative experimental APs (top panels) from Ma et al^1^ and selected simulated APs (bottom panels).

### Symptomatic vs asymptomatic mutant models

**Figure 3:**
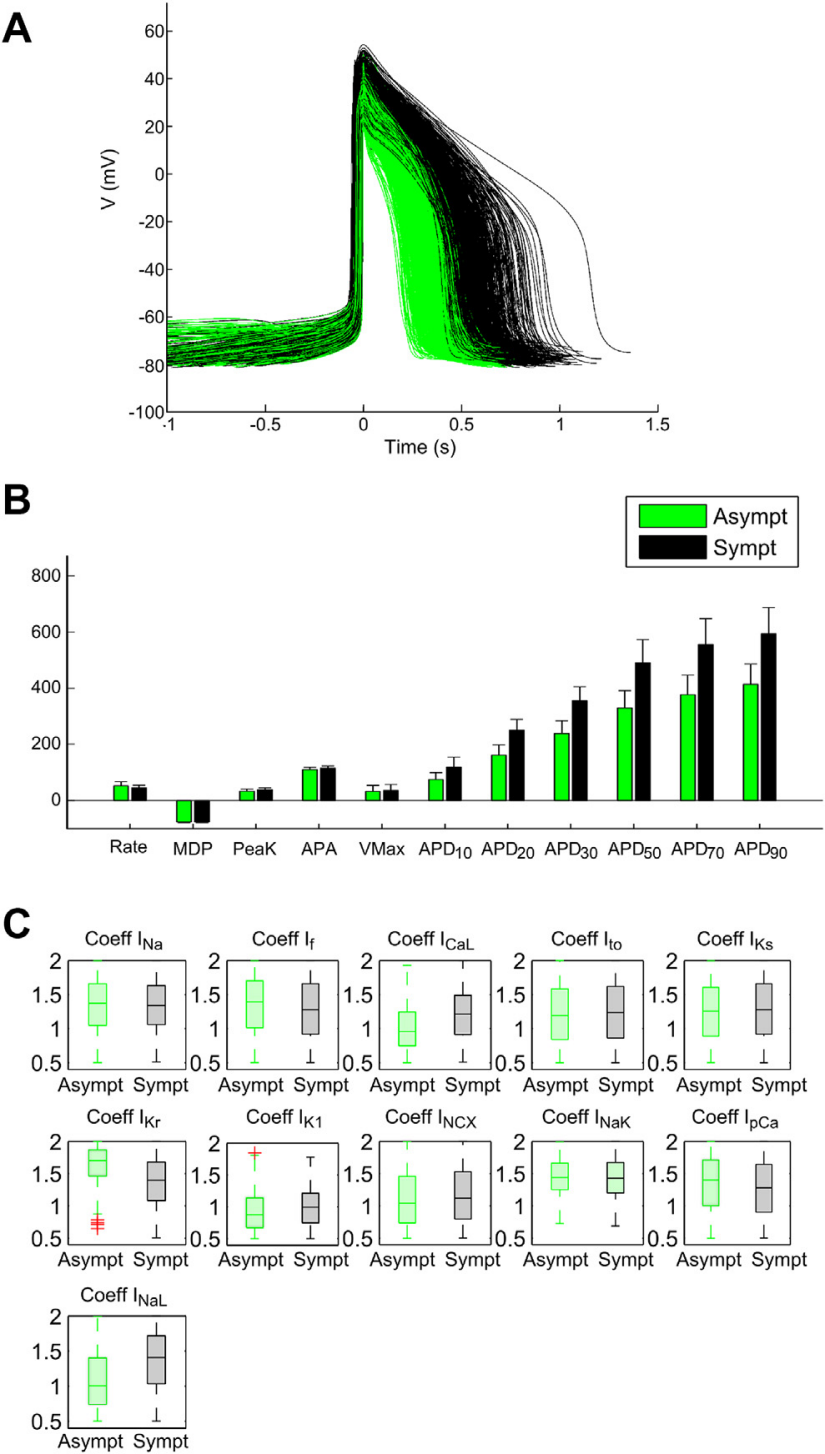
Symptomatic *(black)* vs asymptomatic *(green)* APs **(A)** and biomarkers **(B)** from simulated hiPSC-CMs. **C:** Maximum conductances of symptomatic and asymptomatic models. I_CaL_ = L-type calcium current; I_f_ = hyperpolarization-activated cyclic nucleotide-gated funny current; I_K1_ = the inward rectifying potassium current; I_Kr_ = rapid delayed rectifying potassium currents; I_Ks_ = slow delayed rectifying potassium currents; I_Na_ = fast sodium current; I_NaK_ = sodium-potassium pump; I_NaL_ = late sodium current; I_NCX_ = sodium-calcium exchanger; I_pCa_ calcium sarcolemmal pump; I_to_ = transient outward potassium current. Other abbreviations as in Figure 1 and Figure 2.

The mutant population was split into asymptomatic (with the shortest APDs; n = 678) and symptomatic (n = 723) groups (Figures 3A and 3B). The ionic bases of the phenotypic differences were investigated, highlighting important differences in maximal conductances for I_CaL_, I_Kr_, the inward rectifying potassium current (I_K1_), the calcium sarcolemmal pump (I_pCa_), and I_NaL_ (Figure 3C). In particular, symptomatic hiPSC-CMs models displayed larger I_NaL_ (median 78.3 vs 55.5 S/F, +41.0%), larger I_CaL_ (105 vs 83 cm^3^/F/s, +27.1%), smaller I_pCa_ (0.53 vs 0.58 A/F, –8.6%), and smaller I_Kr_ (41.8 vs 50.8 S/F, –17.7%). Larger I_CaL_ means larger inward current, whereas smaller I_Kr_ and I_pCa_, reduced outward current; these factors resulted in reduced repolarization reserve in mutant symptomatic models. Finally, larger I_K1_ in the symptomatic models (28.1 vs 24.8 S/F, +13.6%) induced greater activation of the Na^+^ currents (see Supplementary Table S5 and Supplementary Figure S4). We finally evaluated whether the control models corresponding to symptomatic vs asymptomatic mutant models displayed differences in biomarkers. The analysis reveals differences in biomarkers between those two control hiPSC-CM groups as shown in Supplementary Figure S5. Control models leading to symptomatic LQT3 models display decreased rate (–12%), increased V_Max_ (+27%), and prolonged APD_90_ (+34%) compared to those leading to asymptomatic LQT3 models after introduction of the I_NaL_ mutation.

### Drug tests

#### Mexiletine

**Figure 4:**
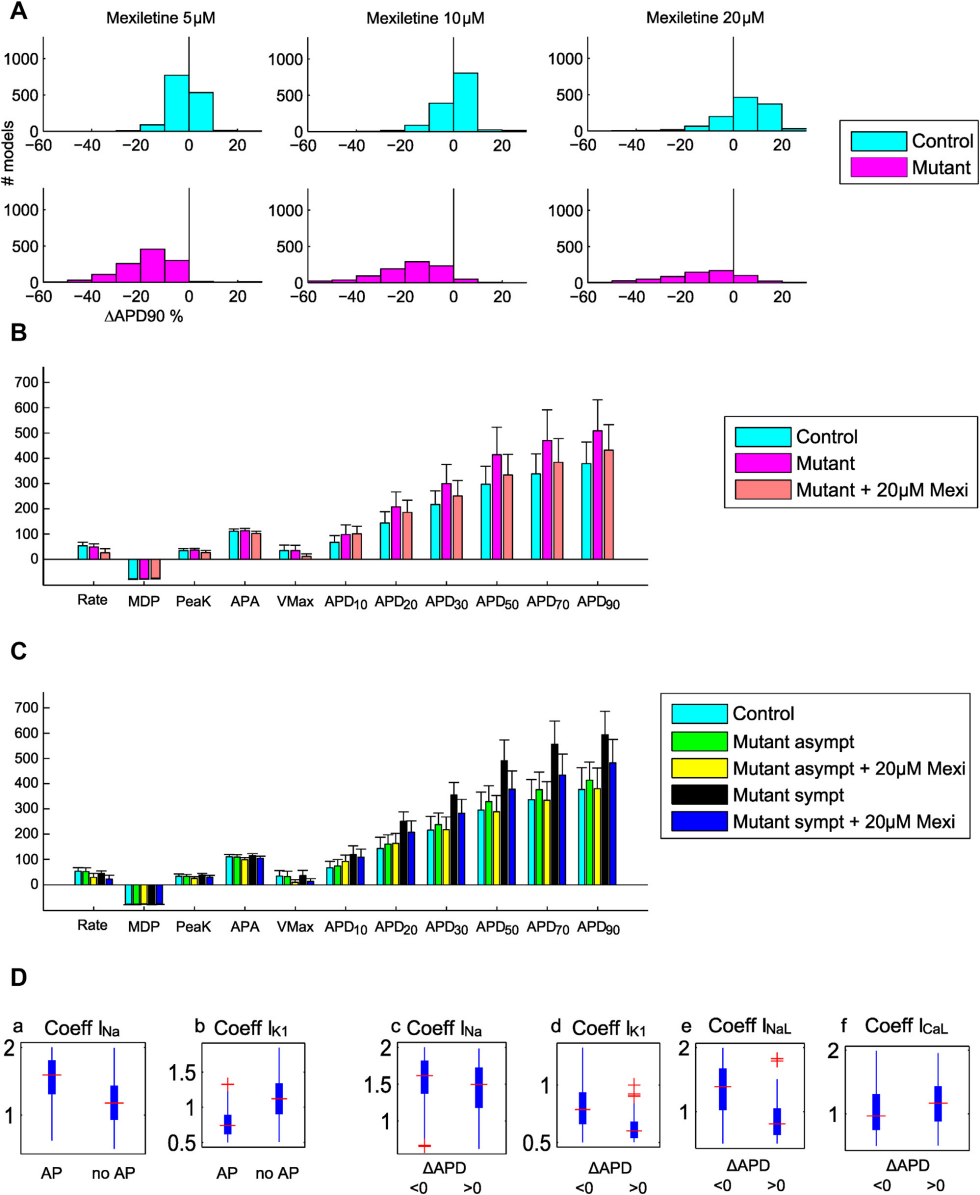
**A:** ΔAPD_90_ for models of control and LQT3 hiPSC-CM populations at 5, 10, and 20 μM mexiletine doses. **B:** Mexiletine effects on AP biomarkers of mutant hiPSC-CM populations. **C:** Mexiletine effects on AP biomarkers of symptomatic and asymptomatic mutant hiPSC-CM models. **D:** Ionic properties underlying phenotypic differences in hiPSC-CM after administration of 20 μM mexiletine. The mutant models producing APs (panels a and b) are then split in panels c–f according to their ΔAPD. I_Na_ is smaller (a) (median 4,331 vs 5,846 S/F, –26%) and I_K1_ is larger (b) (31.5 vs 20.8 S/F, +51%) in models not producing an AP than in those with AP with mexiletine. Models with APD prolongation after mexiletine application have a very weak I_K1_ (d) (16.9 vs 22.1 S/F, –23%), smaller I_NaL_ (e) (44.9 vs 77.2 S/F, –42%), and larger I_CaL_ (f) (101 vs 84 cm^3^/F/s, +21%), with small difference in I_Na_ (c) (5,487 vs 5,927 S/F, –7%). Abbreviations as in Figure 1, Figure 2 and 3.

Figure 4 illustrates the effect of the three doses of mexiletine on the APD of control and mutant populations (further characterized in Supplementary Table S6). Interestingly, because of the different effects of mexiletine on control and mutant Na^+^ currents,^8^ results show slight APD_90_ prolongation in controls (+5.1 ms at 10 μM and +23.5 ms at 20 μM), consistent with experiments by Malan et al^4^ on stimulated hiPSC-CMs, and significant AP shortening (–96.2 ms at 10 μM and –76.6 ms at 20 μM) in the mutant population (Figures 4A and 4B). Due to I_Na_ block by mexiletine (Supplementary Table S3), a dose-dependent amount of models stopped producing APs (216 at 5 μM, 483 at 10 μM, and 784 at 20 μM). The most significant differences between the mutant models producing or not producing APs at 20 μM are the smaller I_Na_ (median 5006 vs 3377 S/F, –33.3%) and the greater I_K1_ (25.6 vs 36.0 S/F, +40.6%) (Figure 4D, panels a and b).

Figure 4C shows the effect of 20 μM of mexiletine on the asymptomatic and symptomatic models from the mutant population. The global effect is APD shortening; APD_90_ was shortened by 111.4 ms (–18.7%) for the symptomatic models and by 33.5 ms (–8.1%) for the asymptomatic models. Figure 4A and Supplementary Table S6 show that a large number of mutant models exhibited APD prolongation when administered a high dose (20 μM) of mexiletine. By comparing the subgroups of models that showed a prolongation or shortening of APD_90_ in response to 20 μM mexiletine (see Supplementary Figure S6), we noted depolarized maximum diastolic potential (MDP) and reduced V_Max_ in the subgroup with positive ΔAPD_90_, which was confirmed by reduced I_K1_ (Figure 4D, panel d, and Supplementary Figure S6, inset), revealing decreased Na^+^ current availability in these cells (illustrative APs in Supplementary Figure S7). Under these conditions, strong block of I_Na_ (as for mexiletine) has a dramatic effect on AP upstroke, delaying it and prolonging APD (Figure 4D, panels c and d). Moreover, during the repolarization phase, the same group showed smaller I_NaL_, that is, the main mexiletine target (–42%), and greater I_CaL_ (+21%) (Figure 4D, panels e and f).

As shown in Section S2.1 of the Supplementary Material (see Supplementary Figures S8 and S9, and Supplementary Tables S7–S9), mexiletine had similar effects on AP shape in simulations with the 10 adult cardiomyocytes models based on the O'Hara-Rudy model (mean ΔAPD_90_ +13% for control and –8% for mutant models). Consistent with the hiPSC-CM simulations, mexiletine exhibited stronger action on symptomatic adult mutant models (mean ΔAPD_90_ = –24%) than in asymptomatic models, in which AP was prolonged (mean ΔAPD_90_ = +7%). This shows that the paradoxical prolongation of APD_90_ occurs mostly in models characterized by reduced I_NaL_ (as in the asymptomatic models).

### Ranolazine

**Figure 5:**
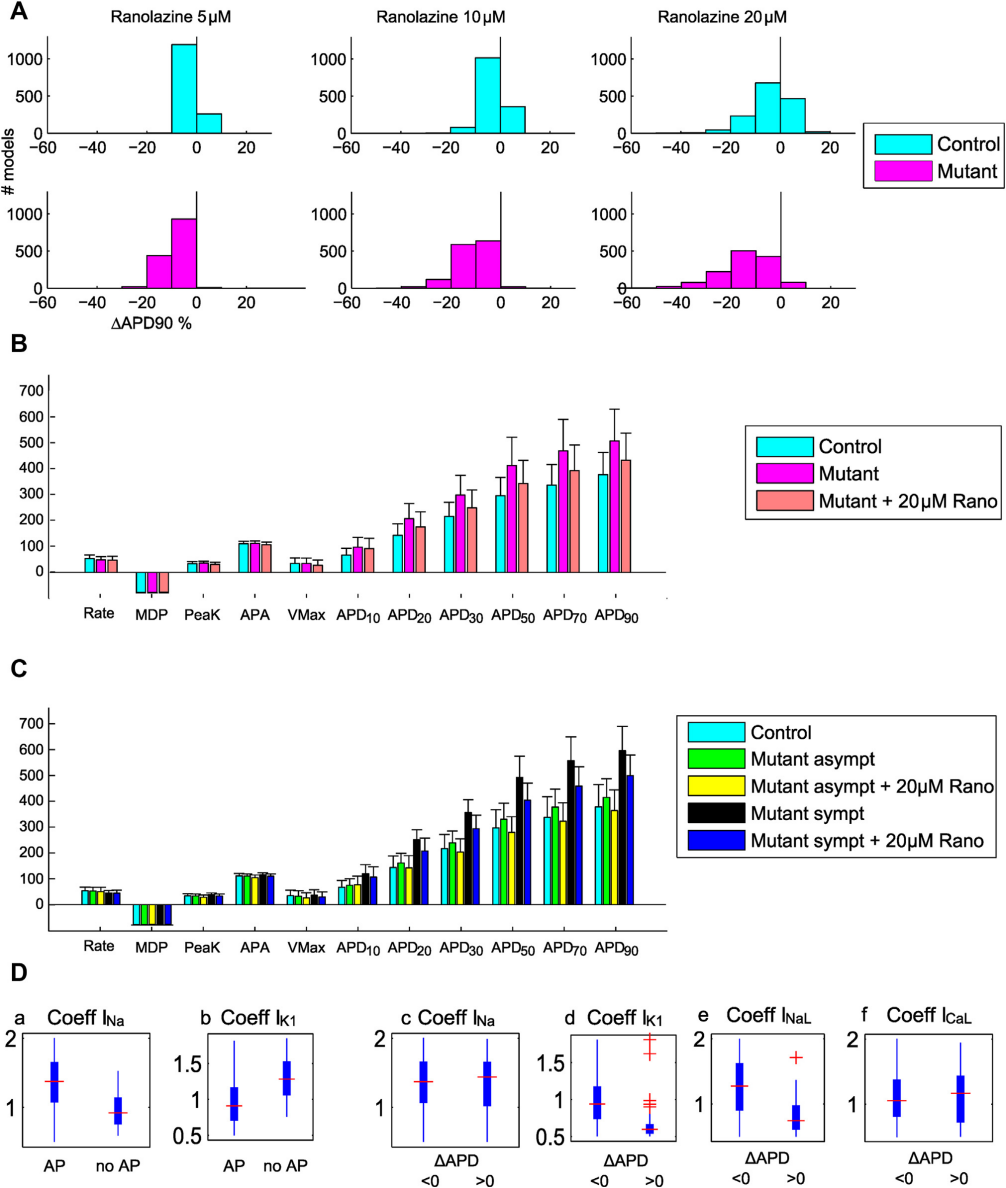
**A:** ΔAPD_90_ for models of control and LQT3 hiPSC-CM populations at 5, 10, and 20 μM ranolazine doses. **B:** Ranolazine effects on AP biomarkers of mutant hiPSC-CM populations. **C:** Ranolazine effects on AP biomarkers of symptomatic and asymptomatic mutant hiPSC-CM models. **D:** Ionic properties underlying phenotypic differences in hiPSC-CM after administration of 20 μM ranolazine. The mutant models producing APs (panels a and b) are then split in panels c–f. I_Na_ is smaller (a) (median 3,384 vs 5,049 S/F, –33%) and I_K1_ larger (b) (36.1 vs 25.9 S/F, +41%) in models not producing an AP than in those with AP with ranolazine. Models with APD prolongation after ranolazine application have a very weak I_K1_ (d) (16.8 vs 26.4 S/F, –37%) and smaller I_NaL_ (e) (41.3 vs 70.8 S/F, –41%) with small differences in I_Na_ (c) (5,286 vs 5,037 S/F, +5%) and I_CaL_ (f) (101 vs 91 cm^3^/F/s, +11%). Abbreviations as in Figure 1, Figure 2 and 3.

Figure 5A shows how ranolazine dragged the APD values of mutant models toward control values. As shown in Figure 5B, APD_90_ in the control and mutant populations was shortened on average –9.6 ms and –57.1 ms at 10 μM, –16.0 ms and –74.1 ms at 20 μM, respectively, and to a greater extent in the mutant group (see Supplementary Table S6). Again, models characterized by large I_K1_ and small I_Na_ stopped producing APs (3 at 5 μM, 12 at 10 μM, and 53 at 20 μM). At 20 μM, the group not producing APs showed greater I_K1_ (+51.35%) and smaller I_Na_ (–25.78%) compared to the group producing APs (Figure 5D, panels a and b). As for mexiletine, in the control population a large number of models exhibited APD_90_ prolongation (see Supplementary Table S6). This phenomenon also affected a limited number of mutant models (11 at 5 μM, 23 at 10 μM, and 83 at 20 μM ranolazine) and to a lesser extent compared to mexiletine. Again, these models show smaller V_Max_ and depolarized MDP as consequence of reduced I_K1_ (Figure 5D, panel d), which affects Na^+^ current availability, thus delaying the upstroke in case of I_Na_ block. Furthermore, these models show smaller I_NaL_ (–42% at 20 μM) compared to the models exhibiting AP shortening (Figure 5D, panel e). For the same dose, ranolazine blocks I_Na_ to a lesser extent than mexiletine, therefore leading to APD_90_ prolongation in fewer (83) models. Finally, Figure 5C shows how 20 μM ranolazine affects the symptomatic and asymptomatic models. The global effect is APD shortening, by 96.5 ms (–16.2%) for the symptomatic models and by 51.23 ms (–12.4%) for the asymptomatic models.

As for mexiletine, also in the human adult APs models, ranolazine showed stronger action on the five symptomatic mutant models (mean ΔAPD_90_ = –12%) than on the five asymptomatic ones (mean ΔAPD_90_ = +8%) (see Supplementary Section S2.1, Supplementary Figures S8 and S9, and Supplementary Tables S7–S9).

Finally, in Section S2.2 of the Supplementary Material, we also report the effect of 100 μM ranolazine (see Supplementary Figure S10 and Supplementary Table S10), which is able to restore the mutant symptomatic APD to its asymptomatic value (422 vs 414 ms).

## Discussion

The present study demonstrates the ability of populations of hiPSC-CMs *in silico* models to simulate and suggest potential mechanisms, which can be further tested in laboratory, underlying experimental variability in AP of control and LQT3 mutant cells observed *in vitro*, as well as their response to two antiarrhythmic blockers used in the treatment of LQT3 syndrome. Additional specific findings include the following: (1) the key properties determining symptomatic vs asymptomatic LQT3 hiPSC-CM phenotypes in the simulations are the magnitude of I_NaL_, in combination with I_CaL_, I_Kr_ and I_K1_, which, depending on their magnitudes, either exacerbate or compensate the mutation effects; (2) variability of mutant hiPSC-CM responses to antiarrhythmic drugs can be reproduced *in silico*, enabling the investigation of likely underlying ionic mechanisms; (3) mexiletine confirmed its efficacy in shortening APD, but in subpopulations it led to termination of the trigger of spontaneous APs or to paradoxical APD prolongation due to slowing of depolarization and reduced weak repolarization, in models with reduced I_NaL_ and large I_CaL_; and (4) ranolazine proved to be as effective as mexiletine in shortening APD and led to AP termination and paradoxical APD prolongation in fewer cells than mexiletine did.

Variability in hiPSC-CMs observed *in vitro* is a known issue, widely demonstrated in the literature (see Supplementary Table S2).^1^, ^2^, ^5^, ^14^, ^15^, ^16^ A canonical *in silico* model, based on average data, can surely provide mechanistic insights on electrophysiological mechanisms; however, it is inadequate to capture and explain variability or its causes. Experimentally calibrated *in silico* populations provide a tool to explore a wide range of ionic scenarios, which enables recapitulation of the variability in experimental recordings and provides plausible explanations for the ionic mechanisms underlying variability in cardiac cell phenotypes. In our study, the control population provided a pool of models covering the experimental biomarker range (see Supplementary Figure S3).^1^, ^2^, ^5^, ^14^, ^15^, ^16^ Analysis shows interesting correlations between AP amplitude (APA) - peak potential (Peak) and V_Max_-MDP (the more negative the MDP, the larger the V_Max_ due to greater I_Na_ availability). This calibrated population of hiPSC-CM was “transfected” with the LQT3 mutation, and our results show agreement with the *in vitro* dataset reported by Ma et al.^1^ Of note, the mutant population was not obtained by direct experimental calibration, but the mutation was expressed in all the models of the experimentally calibrated control population. We chose this approach (as in Passini et al^17^) for the following reasons: (1) this approach allowed an (almost) 1:1 correspondence between control and mutant models, while simultaneously allowing considering a wide range of ionic scenarios; and (2) there is only one experimental dataset for the LQT3 biomarkers^1^ rather than the six datasets available for control hiPSC-CMs. Calibrating the mutant population with 1 dataset only (containing 12 cells) would have dramatically limited the variability in the mutant population.

Our simulations suggest that strong repolarization reserve yields asymptomatic LQT3 models. Indeed, simulations showed that mutant models with normal APs (asymptomatic models) featured smaller inward I_CaL_ and stronger outward I_Kr_ and I_pCa_. Together with a smaller I_K1_, which affects the availability of Na^+^ currents, and the physiological variability of I_NaL_ conductance, such interplay of currents allows compensating for the LQT3 mutation in clinically asymptomatic mutant carriers. Results on the stronger I_pCa_ in particular require further evaluation, because such current usually is not as carefully characterized experimentally as other currents in cardiomyocyte models. We also studied the differences between the control models that became symptomatic after the introduction of the mutation and those that remained asymptomatic. The first group was characterized by a significantly longer AP (APD_90_ +34%). This shows that APs with specific characteristics, and weak repolarization reserve, may indicate symptomatic behavior in case of mutation.

Furthermore, our simulations are the first population-based *in silico* hiPSC-CM drug trial using control and mutant populations to assess in a wide range of ionic scenarios the effect of two drugs used in LQT3 treatments: mexiletine^7^ and ranolazine,^10^ Simulations confirmed the efficacy of mexiletine in shortening the APD in mutant cells, whereas they displayed slight APD_90_ prolongation in controls. These results are consistent with experiments by Malan et al.^4^ However, *in vitro* laboratory recordings are usually conducted on a limited number of cells (order of tens) because of the time and work required for cell culturing and wet lab experiments. As long as computing power and time are available for the simulations (see Supplementary Table S11), *in silico* populations of models can be used to overcome these limitations and thus offer a powerful platform to further evaluate potential effects of mutations and drugs.

An additional finding is that mexiletine inhibited spontaneous APs in a large hiPSC-CM subgroup. At 20 μM, 784 of 1,401 models (56%) stopped producing APs. This behavior was due to a weak I_Na_ (Figure 4D, panels a and b), which could not trigger the upstroke despite I_Na_ activation due to strong I_K1_. Because we considered spontaneous APs, we tested low Na^+^ blocker concentrations, as stronger doses would have inhibited spontaneous APs. Furthermore, in a subgroup of mutant cells producing APs, APD_90_ was prolonged (23, 52, and 132 models at 5, 10, and 20 μM, respectively). Causes included delayed upstroke, due to a weak I_K1_ (impairing I_Na_ activation) (Figure 4D, panels c–f, and Supplementary Figure S6) and a repolarization phase in which I_NaL_ was strongly reduced (median 44.9 vs 77.2 S/F, –42%), that is, it lacked part of the main mexiletine target, and I_CaL_ was increased (101 vs 84 cm^3^/F/s, +21%). Interestingly, the models presenting such prolongation at 20 μM were 91 of 132 (69%) asymptomatic and only 41 of 132 (31%) symptomatic phenotypes, suggesting unexpected mexiletine effects on asymptomatic cells. Although this behavior still requires experimental confirmation, we suggest that it should be considered when testing strong I_Na_ blockers on hiPSC-CMs, especially for spontaneous APs.

Finally, both ranolazine and mexiletine led to APD shortening to a comparable extent. Only a few mutant models (11, 23, and 83 at 5, 10, and 20 μM, respectively) showed APD prolongation, associated with low I_K1_ and I_NaL_ (Figure 5D, panels c–f). The lower occurrence of APD prolongation (58 of 83 symptomatic, 25 of 83 asymptomatic) for ranolazine than mexiletine was due to the smaller impact of ranolazine on I_Na_ (see Supplementary Tables S3 and S6). This suggests that a key factor in the variability of the responses of LQT3 mutant cells to mexiletine and ranolazine is a fine balance between I_Na_, which is responsible for the upstroke phase, I_K1_, which strongly influences I_Na_ availability, and I_NaL_.

Importantly, both drugs were more effective on symptomatic than on asymptomatic hiPSC-CMs, showing greater APD_90_ shortening in the former and confirming their suitability as antiarrhythmic drugs, as confirmed by the simulations with the human adult models. At the same concentration (eg, 20 μM), ranolazine stopped spontaneous electrical activity or induced paradoxical prolongation in fewer hiPSC-CMs than mexiletine did.

### Study limitations

We acknowledge that more detailed modeling approaches to I_Na_, I_NaL_, and LQT3 are available (eg, Moreno et al^19^), and those may be more suitable for detailed studies on arrhythmia mechanisms. In our study, we chose the simpler formulations based on a Hodgkin-Huxley model for the I_NaL_ mutation and a simple pore block model for current–drug interaction based on drug IC_50_ as these are often available for a variety of drugs and ionic currents. Furthermore, an assumption is that variability in ionic conductances is a major determinant of phenotypic variability. Our results demonstrate that this approach is sufficient to reproduce and investigate phenotypic variability in hiPSC-CMs in control and LQT3, and in response to two drugs. Other factors such as ionic current kinetics may make additional contributions (eg, in Britton et al^13^), which could be explored in future studies. It also is possible that ionic backgrounds and phenotypes included in the populations are not observed in experiments and certainly in those used for calibration. This may be an advantage of the *in silico* population, as it allows investigation of a wider range of scenarios than experimentally, potentially highlighting possible phenotypes either not observed (eg, due to limited number of cells) or not reported (eg, termination of spontaneous activity interpreted as degradation of the preparation after drug administration).

## Conclusion

We demonstrated the ability of computer simulations to capture and offer hypotheses for variability in LQT3 and control hiPSC-CM phenotype and in their response to mexiletine and ranolazine. Populations of *in silico* hiPSC-CM models were constructed based on cellular and ionic recordings and were shown to be in agreement with available experimental data for control and LQT3 mutation, and after drug application. The mutant population was divided into symptomatic and asymptomatic models based on their AP characteristic. The symptomatic LQT3 hiPSC-CM models display weaker repolarization reserve compared to LQT3 hiPSC-CM with normal APD, primarily determined by I_NaL_, I_CaL_, I_Kr_, and I_K1_. Both mexiletine and ranolazine were shown to be generally effective in shortening APD of LQT3 hiPSC-CMs, with additional cell subgroups responding with both AP suppression and prolongation. Our results highlight the power of *in silico* populations of models in exploring phenotypic hiPSC-CM variability, thus supporting compound prescreening and aiding in the interpretation of *vitro* drug studies.
