# Supplementary material for: Phenotypic variability in LQT3 human induced pluripotent stem cell-derived cardiomyocytes and their response to antiarrhythmic pharmacologic therapy: An in silico approach
Source: Heart Rhythm. 2017 Nov;14(11):1704–12. doi: 10.1016/j.hrthm.2017.07.026 (PMC5668441; doi:10.1016/j.hrthm.2017.07.026)
Supplement: Supplementary Material [file mmc1.pdf]

Supplementary material

Phenotypic variability in LQT3 human induced pluripotent stem cell-derived cardiomyocytes and their response to anti-arrhythmic pharmacological therapy: an in silico approach

Michelangelo Paci, Elisa Passini, Stefano Severi, Jari Hyttinen, and Blanca Rodriguez

S1. Supplementary methods

S1.1 Baseline and LQT3 models

The late sodium current (I_{NaL}) formulation was incorporated in the Paci2015 hiPSC-CM AP model[1,2], thus enabling the simulation of LQT3 V1763M mutation. The I_{NaL} formulation was based on the one proposed by O'Hara-Rudy[3], increasing its maximum conductance by 2.3 to fit the control value[4] 0.65±0.11 pA/pF. The V1763M I_{NaL} mutation was simulated by i) increasing by 7.4 the maximum conductance and ii) tripling the inactivation time constant, to fit the experimental value[4] of 3.16±0.27 pA/pF. In spite of the availability of more complex and detailed models, we chose Hodgkin & Huxley formulations to simulate the control and mutant Na^+ currents: Carbonell-Pascual et al.[5] showed that at least for the mutations of I_{Kr} and I_{Na} they evaluated, simpler Hodgkin & Huxley current models produce simulations equivalent to those obtained with more complex Markovian models, in terms of action potentials (which is the focus of our paper) and also in terms of arrhythmic markers. Furthermore, we

evaluated the possibility of including the small conductance calcium-activated potassium current (I_{SK}) in the model, shown to be important in adult ventricular cardiomyocyte repolarization in a previous *in silico* study[6]. Since, to our knowledge, no I_{SK} measurements on hiPSC-CMs were published, we tested the effect on the APD of including the adult I_{SK} formulation, which in the study by Kennedy et al.[6] shortened APD_{90} by 12%. In both our baseline and LQT3 models, I_{SK} accounted only for a 1% APD_{90} shortening, and doubling I_{SK} maximum conductance (1.6 instead of 0.8 µS/µF) shortened APD_{90} by less than 2%. Given the small effect on APD and the lack of I_{SK} evidence in hiPSC-CM, we did not retain I_{SK} in the models used to generate the populations. Specific measurements are needed for further investigations into the potential role of I_{SK} on hiPSC-CMs repolarization. Model equations and parameter changes (Table S1) are reported in the Supporting Information.

S1.2 I_{NaL} equations and parameters

Control I_{NaL} data were taken from Ma[4], where I_{NaL} was defined as the persistent current component recorded 200 ms after stimulus from -90mV to -10mV, resulting in 0.65±0.11 pA/pF. The I_{NaL} formulation was based on the one proposed by O'Hara-Rudy[3], increasing I_{NaL} maximum conductance by 2.3 to fit such value. In Ma[4] the LQT3 mutation was reported to increase I_{NaL} up to 3.16±0.27 pA/pF. The V1763M mutation I_{NaL} formulation was again based on the O'Hara-Rudy formulation by i) increasing by 7.4 the maximum conductance and ii) tripling I_{NaL} inactivation time constant, to fit the experimental value of 3.16±0.27 pA/pF at 200 ms from stimulus (see Table S1).

Table S1. Parameter changes induced by the LQT3 mutation in the wild type and mutant baseline models.

	Adult [3]	Control hiPSC-CM	LQT3 hiPSCM
I_{NaL}			
G_{NaL} (pA/pF)	7.5	2.3 × 7.5	7.4 × 7.5
SS inactivation τ (ms), tauINaL	200	200	3 × 200

```
% Vm:   membrane potential (mV)
% E_na: sodium Nernst potential
m_inf_L = 1/(1+exp(-(Vm+42.85)/(5.264)));
alpha_m_L = 1/(1+exp((-60-Vm)/5));
beta_m_L = 0.1/(1+exp((Vm+35)/5))+0.1/(1+exp((Vm-50)/200));
tau_m_L =alpha_m_L*beta_m_L;
dm_L = (m_inf_L-m_L)/tau_m_L;

h_inf_L = 1/(1+exp((Vm+87.61)/(7.488)));
tau_h_L =tauINaL;
dh_L = (h_inf_L-h_L)/tau_h_L;

i_NaL = GNaLmax* m_L^(3)*h_L*(Vm-E_Na);
```

S1.3 Control population of hiPSC-CM models

To investigate hiPSC-CM phenotypical variability under control conditions, a random population of hiPSC-CM control models was developed as proposed in Britton[7]. The updated Paci2015 model with I_{NaL} formulation was used as baseline. Eleven ionic conductances/permeabilities were sampled using the Latin Hypercube method[7] to generate 10000 candidate models sharing the same equations as in the baseline model, but with conductances randomly sampled from 50% to 200% of their original value. The sampled ionic conductances and permeabilities were those of the fast sodium current (I_{Na}), I_{NaL}, the L-type calcium current (I_{CaL}), the hyperpolarization-activated cyclic nucleotide-gated funny current (I_f), the inward rectifying potassium current (I_{K1}), the rapid and slow delayed rectifying potassium currents (I_{Kr} and I_{Ks}), the transient outward potassium current (I_{to}), the sodium-calcium exchanger (I_{NCX}), the sodium-potassium pump (I_{NaK}) and the calcium sarcolemmal pump (I_{pCa}). Simulations were run for each parameter set for 800 s to reach the steady state, with no external stimulation, i.e. considering the hiPSC-CM spontaneous APs.

S1.4 Calibrating the random population and obtaining the control population

The random population was then calibrated to select models in range with experimental data. Six hiPSC-CM AP datasets were considered (Ma2011[8], Moretti2010[9], Ma2013[4], Fatima2013[10], Lahti2012[11] and Kujala2012[12]). In Table S2, we summarized the biomarkers, including: i) rate of spontaneous AP (Rate), ii) maximum diastolic potential (MDP), iii) peak potential (Peak), iv) AP amplitude (APA), v) maximum upstroke velocity (VMax) and vi) AP duration at different repolarization percentages

(APD_{xx}). For each biomarker, we considered the union of the sets mean±2STD from each dataset (hypothesizing a normal distribution) thus getting the lower (LB) and upper (UB) bounds in Table S2, and the [LB, UB] ranges to which the biomarkers computed on the APs of the random population must belong to. In case of meaningless bounding values, e.g. negative APD, the respective LB was set to zero. As in Britton[7], the calibrated control population contains only those models whose steady state APs have all the biomarkers included in the ranges defined in Table S2. In addition, we introduced two additional calibration criteria: i) we retained only those models able to generate spontaneous APs; ii) the Na^+ and sarcoplasmic Ca^{2+} concentrations were imposed in the following ranges $5 \leq [Na^+]_i \leq 15$ mM and $0 \leq [Ca^{2+}]_{SR} \leq 5$ mM.

Table S2. Datasets used for the first population calibration. Lower (LB) and upper (UB) bounds for each biomarker are reported in bold. Missing data are marked as ---.

**Dataset (# cells)**			**Ma2011 (32)**		**Moretti2010 (21)**		**Ma2013 (12)**		**Fatima2013 (6)**		**Lahti2012 (13)**		**Kujala2012 (16)**	
Biomarker	LB	UB	μ	σ	μ	σ	μ	σ	μ	σ	μ	σ	μ	σ
Rate (bpm)	**>0**	**209**	35	12	68	12	69	39	118	45	72	22	41	24
MDP (mV)	**-89**	**-44**	-76	7	-64	10	-61	5	-64	6	-63	5	-68	7
Peak (mV)	**17**	**58**	28	6	44	7	---	---	39	3	---	---	---	---
APA (mV)	**76**	**139**	104	6	108	10	86	5	102	5	113	9	118	10
VMax (V/s)	**-27**	**82**	28	27	9	1	13	16	24	12	27	23	---	---
APD_{10} (ms)	**20**	**128**	74	27	---	---	---	---	---	---	---	---	---	---
APD_{20} (ms)	**>0**	**290**	---	---	---	---	138	76	---	---	---	---	---	---
APD_{30} (ms)	**59**	**301**	180	61	---	---	---	---	---	---	---	---	---	---
APD_{50} (ms)	**>0**	**601**	---	---	323	139	338	115	175	106	265	54	204	81
APD_{70} (ms)	**146**	**631**	---	---	---	---	388	121	---	---	---	---	---	---
APD_{90} (ms)	**1**	**705**	415	123	381	162	434	108	298	148	314	63	330	90

S1.5 From the control to the mutant population

The control population obtained from the previous experiment-based calibration was then used to generate the LQT3 mutant population. The V1763M mutation I_{NaL} formulation was incorporated in all the models included in the control population (i.e. no calibration was done on the mutant population, following the approach used by Passini[13]) and new simulations were performed (800 s until steady state). Mutant models that did not show spontaneous APs or whose concentrations were out of the ranges $5 \leq [Na^+]_i \leq 15$ mM and $0 \leq [Ca^{2+}]_{SR} \leq 5$ mM were excluded from the analysis.

Due to the lack of an accepted criterion to separate symptomatic and asymptomatic APs, models in the mutant population were classified as asymptomatic and symptomatic using k-means clustering[14] applied to Rate and all the APD (i.e. to the biomarkers reported as statistically different versus control in Ma[4]). Biomarkers were normalized [0, 1] and k-means was repeated 200 times to stabilize the centroids in the biomarker space.

S1.6 Drug tests

Effects of mexiletine and ranolazine at 5, 10 and 20 µM doses were assessed *in silico* on the control and mutant populations considering their multichannel effects on I_{Na}, I_{NaL}, I_{Kr} and I_{CaL} as obtained in ion channel assays and reported in Table S3. Thus, for each current, a single pore block model was used as:

$$\frac{I[D]}{I} = \frac{1}{1+\left(\frac{[D]}{IC_{50}}\right)^H}$$

where *[D]* is the drug concentration, IC_{50} is the drug concentration inducing a 50% current block and *H* the Hill's coefficient. For each model of the control and mutant populations simulations were run for 300 s starting from the model's steady state. We discarded from the analysis the models which did not produce spontaneous APs, or whose intracellular concentrations went beyond the ranges $3 \leq [Na^+]_i \leq 15$ mM and $0 \leq [Ca^{2+}]_{SR} \leq 5$ mM. Due to the smaller intake of Na^+ consequent to the administration of Na^+ channel blockers, we lowered empirically by 2 mM the lower bound for $[Na^+]_i$. An example of the drug effects on the Na^+ currents is reported in Figure S1 and S2. In order to compare the effect of drug action on hiPSC versus adult cardiomyocytes, simulations were conducted considering the same drug doses

on 10 illustrative control and mutant models of human adult ventricular cardiomyocytes, based on the O'Hara-Rudy model[3] (see Section “Comparison with human adult cardiomyocytes” models in the supporting information).

Table S3. Mexiletine and ranolazine IC_{50} and Hill’s coefficients for the four currents considered in this study.

		Mexiletine		Ranolazine	
		CONTROL	MUTANT	CONTROL	MUTANT
I_{Na}	IC_{50} (µM)	43.0[15]	13.3[15]	165.2[16]	120.8[16]
	H	1	1	1.623[16]	1.115[16]
I_{NaL}	IC_{50} (µM)	I_{NaL} 20-fold more sensitive than I_{Na} [15]	2.6[15]	6[16]	12.66[16]
	H	1	1	1	0.7301
I_{Kr}	IC_{50} (µM)	50[17]	50[17]	50[16]	50[16]
	H	1	1	1	1
I_{CaL}	IC_{50} (µM)	100[17]	100[17]	50[18]	50[18]
	H	1	1	1	1

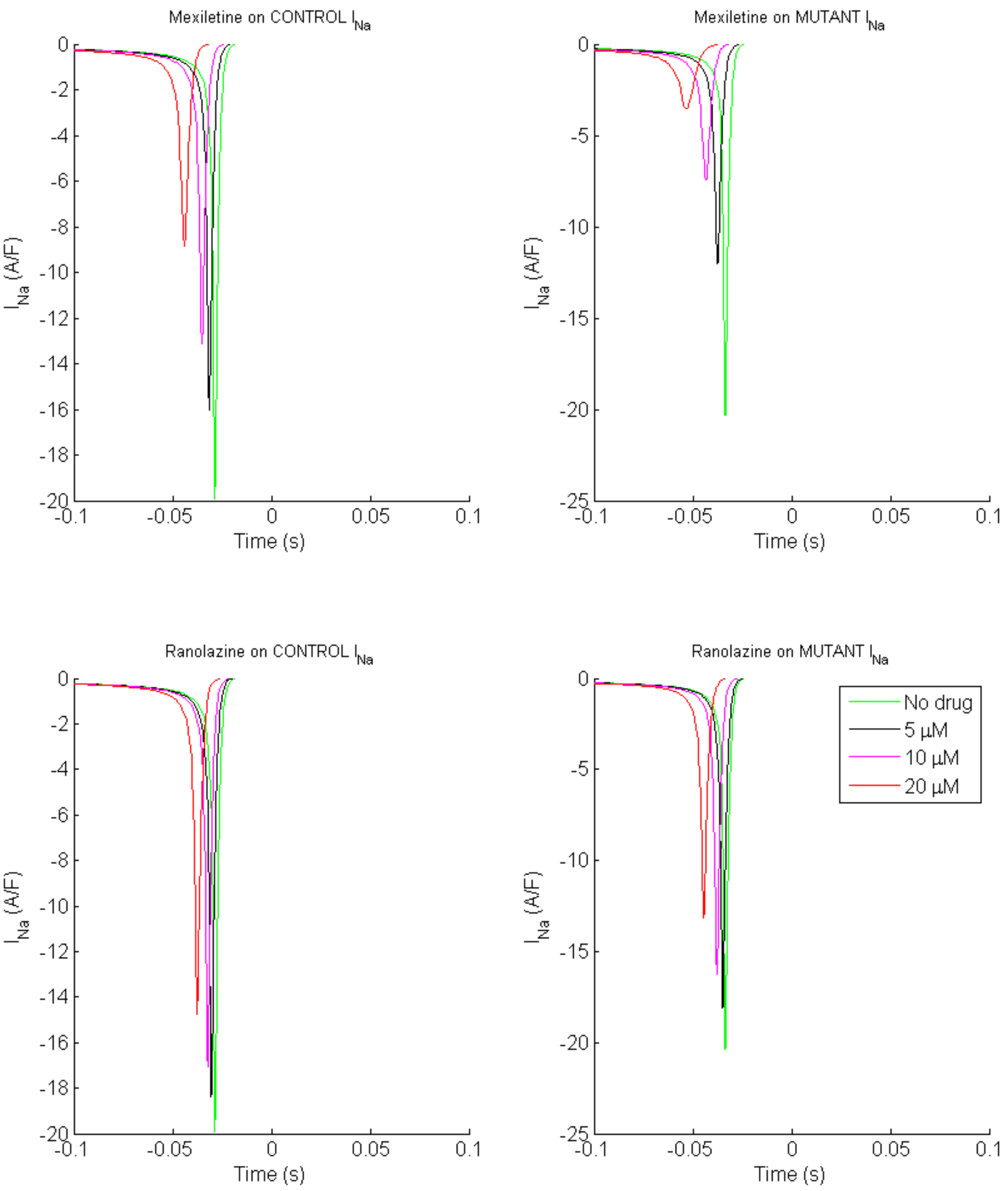

Figure S1. Effects of mexiletine and ranolazine effects on I_{Na} in an illustrative control and mutant hiPSC-CM.

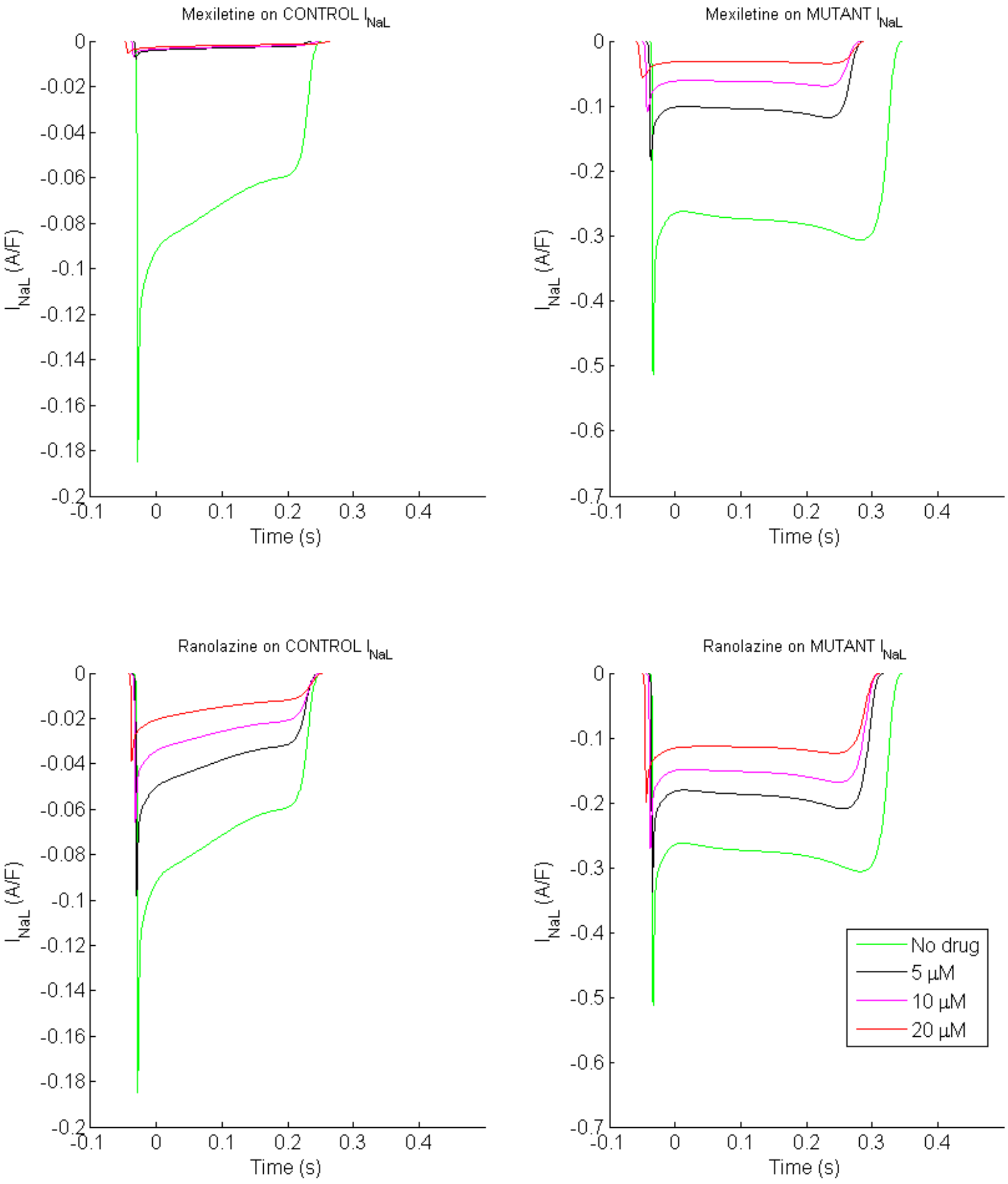

Figure S2. Effects of mexiletine and ranolazine effects on I_{NaL} in an illustrative control and mutant hiPSC-CM.

S2. Supplementary results

Table S4. Action potential biomarkers for the control and mutant baseline hiPSC-CM models, compared to the original ventricular-like biomarker dataset by Ma[8], which was used to develop the Paci2015 model.

Biomarker	Experimental ventricular-like biomarkers [8]	Control hiPSC-CM	Mutant hiPSC-CM
Rate (bpm)	35.3±2.2	37.5	35.1
MDP (mV)	-75.6±1.2	-77.1	-77.1
Peak (mV)	28.3±1.0	28.1	30.3
APA (mV)	104±1	105.3	107.4
VMax (V/s)	27.8±4.8	26.6	26.1
APD_{10} (ms)	74.1±4.8	48.6	86.3
APD_{30} (ms)	180±11	224.0	338.4
APD_{50} (ms)	---	322.9	489.4
APD_{70} (ms)	---	376.1	552.8
APD_{90} (ms)	415±22	416.2	595.7

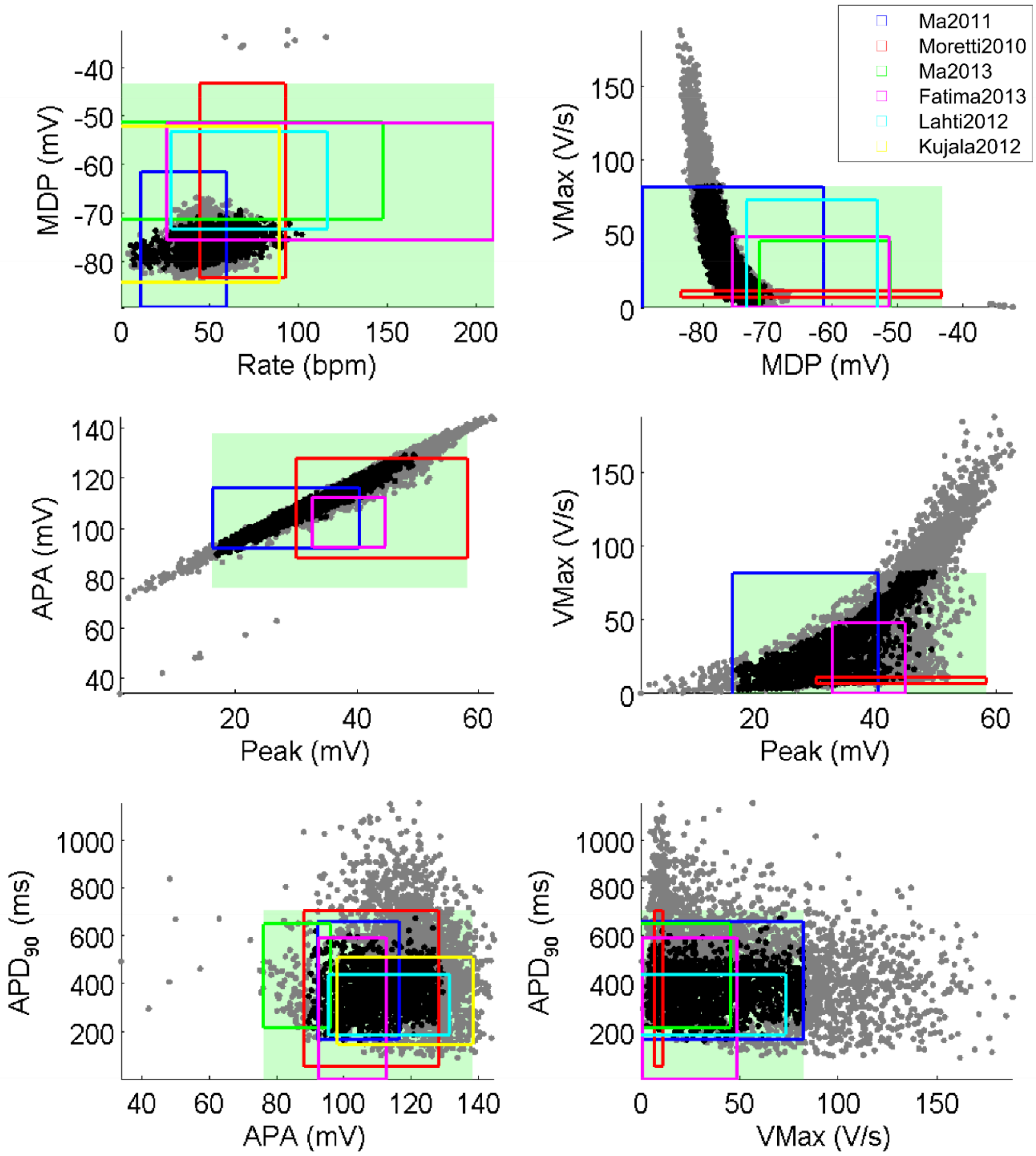

Figure S3. Scatter plots showing biomarkers' values for all the hiPSC-CM models (accepted – black dots; rejected – grey dots). Each dataset is here represented as a colored rectangle. The filled green rectangle represents the overall space of the acceptable biomarker values. Because some biomarkers do not present all the biomarkers here considered (e.g. the dataset Lahti2012 lacks peak voltage), not all the colored rectangles are present in all the panels. The control population successfully covers the experimental biomarker spaces, with the exception of RATE. Actually, RATE values of 150-200 bpm for spontaneous APs were not observed in control conditions on hiPSC-CMs, but for consistency

reasons we generated the range of variability of RATE the same way as for the other biomarkers, i.e. by the union of the single $\mu \pm 2\sigma$ ranges reported in literature

Table S5. I_{K1} coefficients, MDP values, I_{Na} and I_{NaL} peaks for six mutant symptomatic and asymptomatic models. These parameter sets are examples aimed to show the differences due to I_{K1} on the Na^+ currents, reported in Figure S4.

	Model ID	I_{K1} coefficient	MDP (mV)	I_{Na} peak (A/F)	I_{NaL} peak (A/F)
Sympt	4349	1.7808	-78.87	-79.86	-0.5578
	6369	1.7500	-79.88	-77.2	-0.5941
	6232	1.675	-81.36	-66.91	-0.7035
Asympt	4264	0.5013	-72.12	-13.3	-0.1788
	9432	0.5022	-74.73	-20.36	-0.1684
	7635	0.5495	-70.65	-14.57	-0.1619

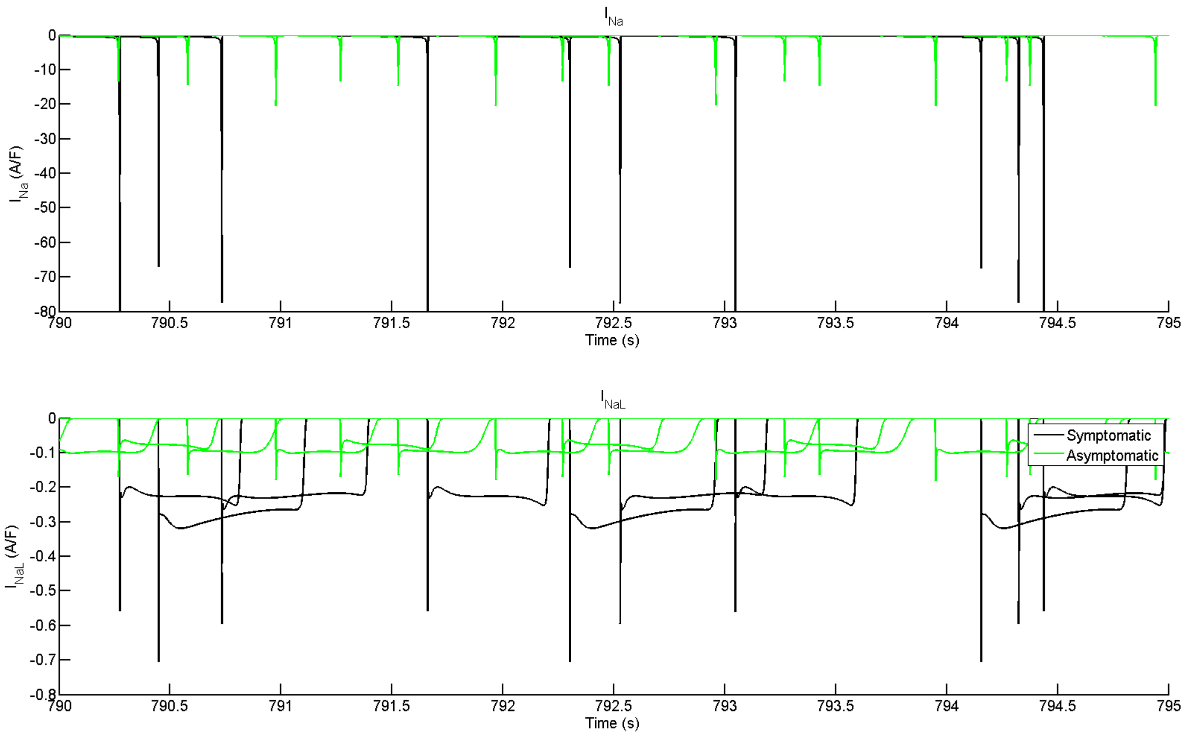

Figure S4. I_{Na} and I_{NaL} differences between three illustrative mutant symptomatic models and three asymptomatic ones, in relation to the overexpression (symptomatic) or underexpression (asymptomatic) of I_{K1}. Specific parameters are reported in Table S5.

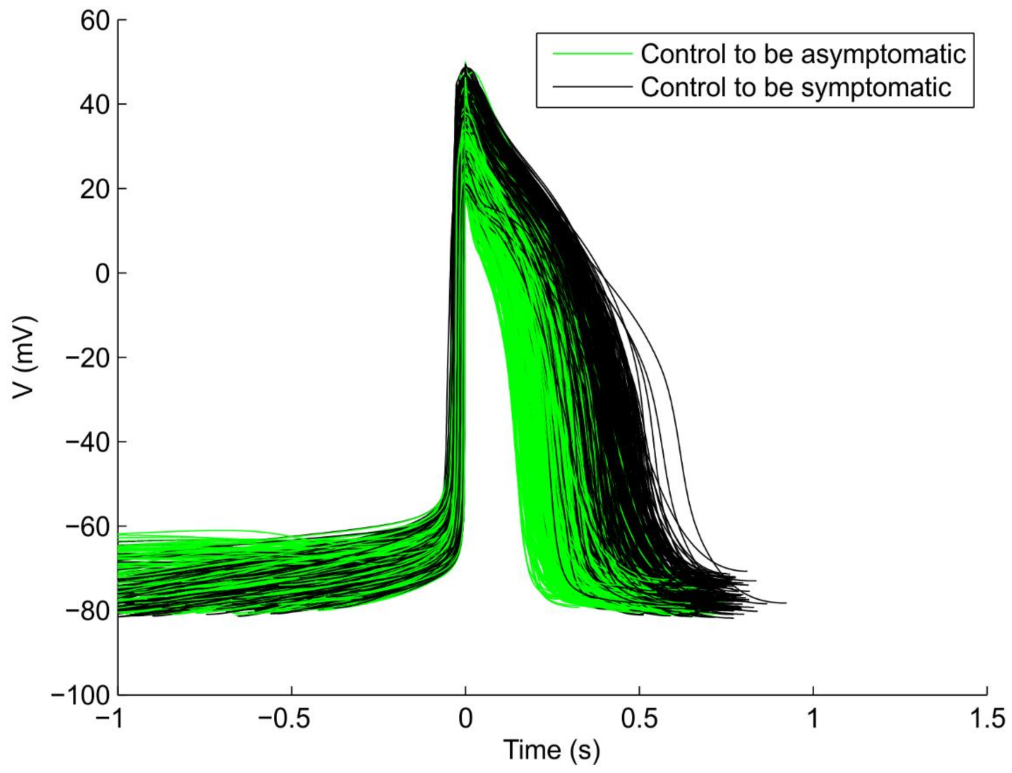

Figure S5. Action potentials of control hiPSC-CM models separated according to the symptomaticity or asymptomaticity when the mutation is introduced.

Table S6. Mean ΔAPD_{90} and number of models with positive and negative ΔAPD_{90} for the control and mutant populations in response to different mexiletine and ranolazine concentrations.

		Mexiletine			Ranolazine		
		mean ΔAPD_{90} (ms)	#models $\Delta APD_{90}<0$	#models $\Delta APD_{90}>0$	mean ΔAPD_{90} (ms)	#models $\Delta APD_{90}<0$	#models $\Delta APD_{90}>0$
5 µM	CONTROL	7.1	867	544	-7.8	1198	258
	MUTANT	-85.2	1162	23	-43.3	1387	11
10 µM	CONTROL	5.1	492	842	-9.6	1094	358
	MUTANT	-96.2	866	52	-57.1	1366	23
20 µM	CONTROL	23.5	293	866	-16.0	964	486
	MUTANT	-76.6	485	132	-74.1	1265	83

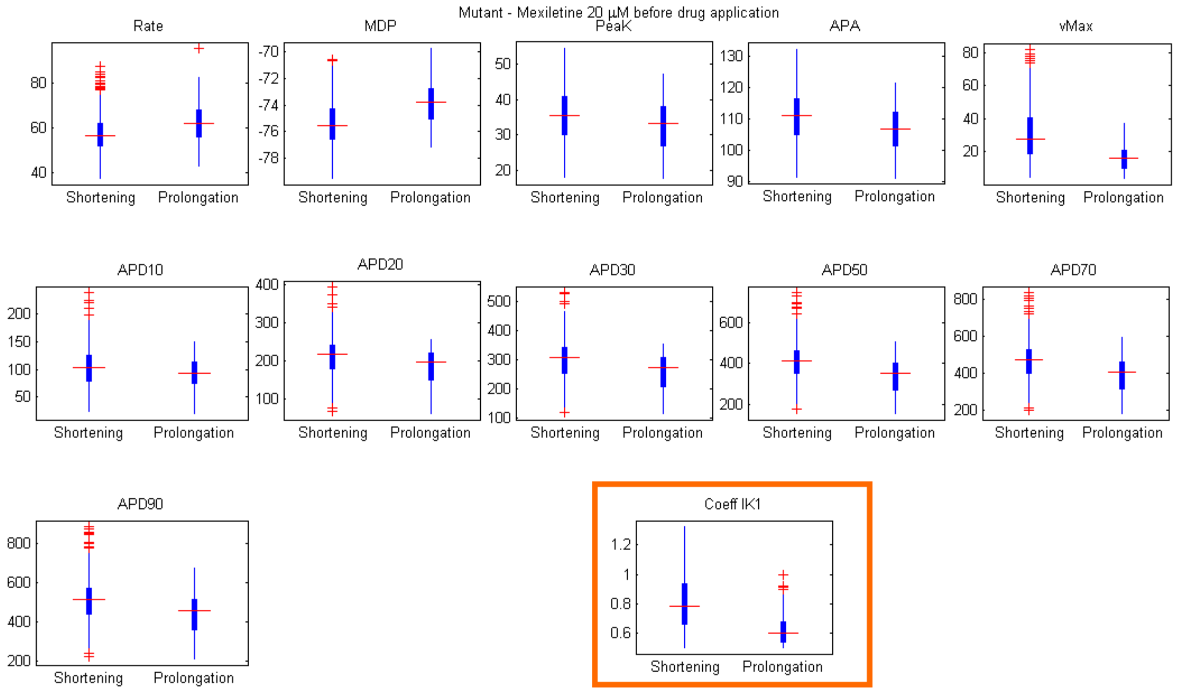

Figure S6. Action potential biomarkers of the mutant hiPSC-CM models (before mexiletine application) which showed a shortening or prolongation of APD_{90} in response to 20 µM mexiletine. The inset shows the smaller I_{K1} conductance for the group characterized by APD prolongation.

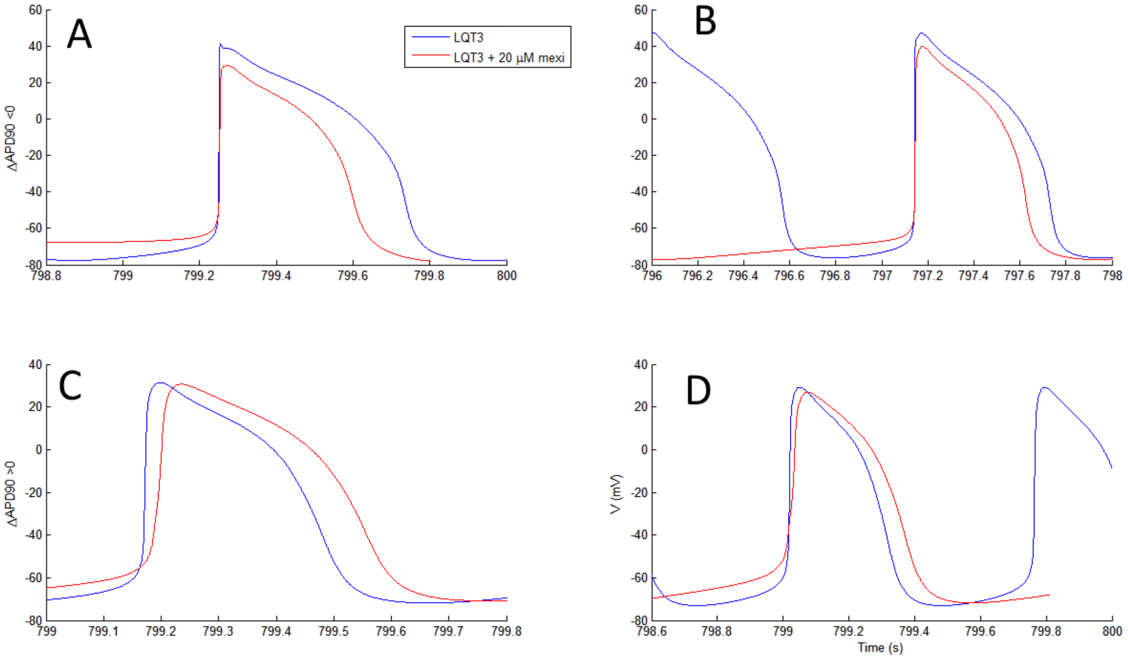

Figure S7. Illustrative APs before and after the administration of 20 µM mexiletine, which induce APD_{90} shortening (panels A and B) or prolongation (panels C and D).

S2.1 Comparison with human adult cardiomyocytes models

We first isolated five symptomatic and five asymptomatic parameter sets (Table S7) and applied them to the O'Hara-Rudy (ORd) model of adult ventricular cell[3]. Symptomatic models were thus characterized with increased I_{NaL}, I_{K1} and I_{CaL}.

Table S7. Parameter sets used to generate the adult models. Coefficients are reported only for I_{Na}, I_{CaL}, I_{K1} and I_{NaL}.

	Model ID	I_{Na}	I_{CaL}	I_{K1}	I_{NaL}
**Sympt**	**1**	**1.00**	**1.00**	**1.00**	**1.00**
	**662**	**1.55**	**1.53**	**1.51**	**1.88**
	**3278**	**1.49**	**1.68**	**1.56**	**1.86**
	**4509**	**1.15**	**1.54**	**1.64**	**1.68**
	**6167**	**1.78**	**1.64**	**1.54**	**1.86**
Asympt	170	1.17	0.57	0.62	0.57
	2793	0.51	0.71	0.62	0.61
	3441	0.95	0.53	0.53	0.58
	4369	1.06	0.65	0.66	0.57
	9691	0.58	1.45	0.68	0.63

We then tested the three concentrations for mexiletine and ranolazine using the same protocol used for hiPSC-CMs. We observed consistency in the drug effects observed in the hiPSC-CM populations and in the illustrative adult models. The following Figures S8 and S9 show the effects of the three concentrations of mexiletine and ranolazine on one symptomatic and one asymptomatic human adult AP models: both drugs shorten or have negligible effects on APD_{90} at 5 and 10 μM in both mutant symptomatic and asymptomatic adult models. At 20 μM dose, APD shortening happens for the symptomatic models with large I_{CaL}, I_{K1} and the mutant I_{NaL} coefficients (mean ΔAPD_{90}:-24% and -12% for mexiletine and ranolazine respectively). In the asymptomatic models with smaller I_{NaL} (i.e. the main target of the drugs), 20 μM induced a slight increase in APD_{90} (mean ΔAPD_{90}= +7% and +8% for mexiletine and ranolazine, respectively). This shows also that the paradoxical prolongation of APD_{90} happens mostly in models characterized by a reduced I_{NaL} (as in the asymptomatic models) and increased I_{CaL}. In contrast with our findings in hiPSC-CMs, we did not observe a distortion of the upstroke phase in the mutant ORd models, when observing the paradoxical effect, but only a reduction

in the AP amplitude. This is due to two reasons: i) the ORd model has a very stable MDP, which is not significantly affected by I_{K1} reduction and ii) the adult simulations required an external stimulus to trigger the APs. In hiPSC-CMs a smaller I_{K1} has been observed compared to adult cells[19] and we studied spontaneous APs, in which the upstroke is more sensitive to variations of I_{Na}.

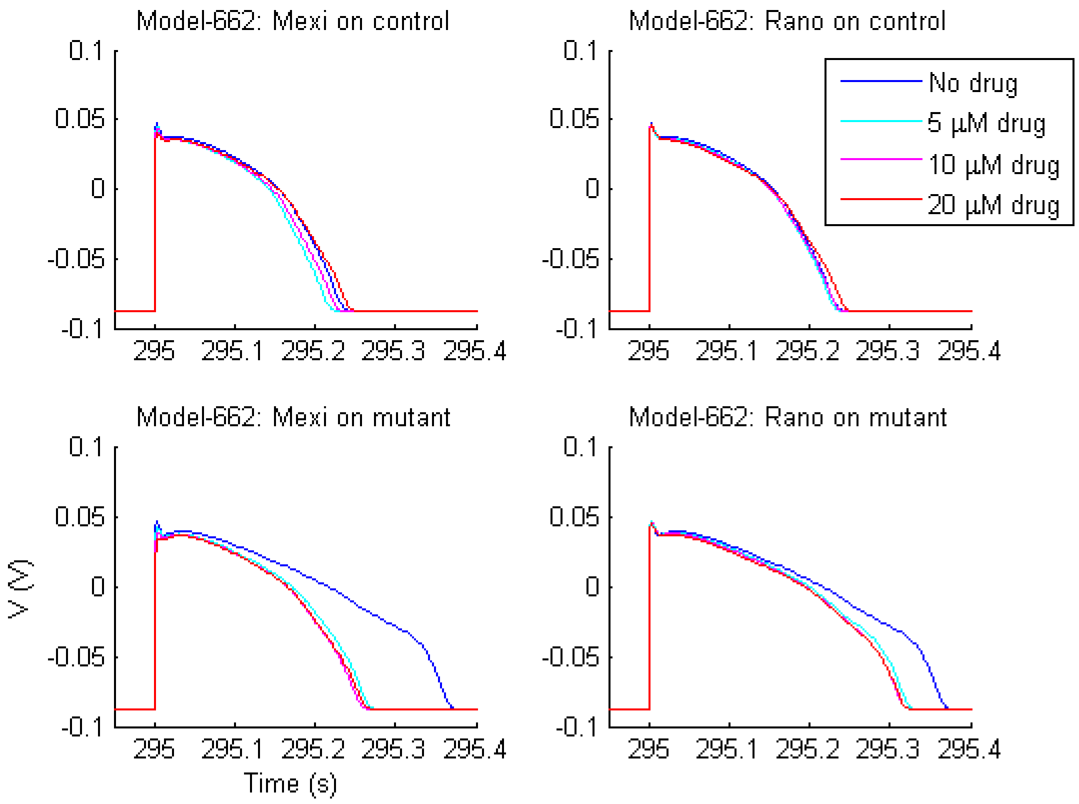

Figure S8. Drug effect on the AP of an illustrative symptomatic adult model (parameter set 662).

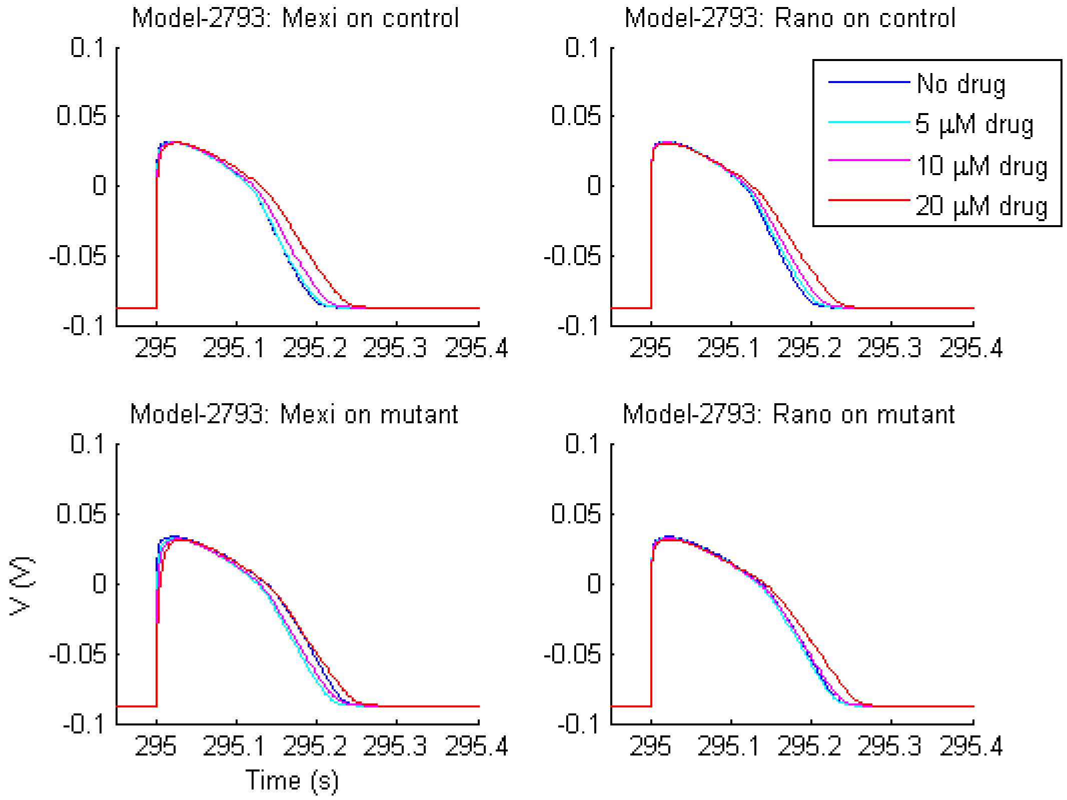

Figure S9. Drug effect on the AP of an illustrative asymptomatic adult model (parameter set 2793).

The following Tables S8 and S9 show the quantification of the AP shortening or prolongation due to drug administration.

Table S8. Mean ΔAPD_{90} induced by different doses of mexiletine and ranolazine. We reported the ΔAPD_{90} both in ms and as percent of variation.

Drug	Dose	ΔAPD_{90} (ms / %)					
		Control			Mutant		
		Total	Asympt	Sympt	Total	Asympt	Sympt
Mexiletine	5 µM	-6 / -2	+3 / +2	-15 / -6	-54 / -16	-14 / -6	-94 / -25
	10 µM	+5 / +3	+15 / +8	-4 / -2	-53 / -14	-5 / -2	-102 / -26
	20 µM	+25 / +13	+36 / +20	+15 / +6	-39 / -8	+15 / +7	-94 / -24
Ranolazine	5 µM	+1 / +1	+5 / +3	-4 / -1	-22 / -6	-3 / -1	-42 / -11
	10 µM	+6 / +3	+13 / +7	-1 / -1	-23 / -6	+3 / +1	-49 / -12
	20 µM	+18 / +9	+29 / +15	+8 / +3	-15 / -2	+18 / +8	-48 / -12

Table S9. APD_{90} changes induced by mexiletine and ranolazine for each of the control and mutant adult models (symptomatic in bold/gray).

Dose	Model ID	ΔAPD_{90} (ms)			
		Mexiletine		Ranolazine	
		Control	Mutant	Control	Mutant
**5 µM**	**1**	**-7**	**-63**	**+1**	**-25**
**10 µM**		**+7**	**-64**	**+6**	**-26**
**20 µM**		**+29**	**-50**	**+19**	**-18**
5 µM	170	+2	-16	+5	-6
10 µM		+16	-7	+12	0
20 µM		+40	+14	+28	+14
**5 µM**	**662**	**-17**	**-101**	**-3**	**-45**
**10 µM**		**-8**	**-110**	**0**	**-52**
**20 µM**		**+8**	**-106**	**+9**	**-51**
5 µM	2793	+4	-15	+6	-1
10 µM		+15	-8	+15	+5
20 µM		+35	+12	+32	+22
**5 µM**	**3278**	**-16**	**-93**	**-5**	**-44**
**10 µM**		**-6**	**-97**	**-2**	**-51**
**20 µM**		**+13**	**-86**	**+7**	**-49**
5 µM	3441	+5	-13	+6	-4
10 µM		+16	-2	+14	+3
20 µM		+39	20	+29	+17
5 µM	4369	+2	-15	+5	-5
10 µM		+16	-5	+13	+1
20 µM		+39	+20	+28	+17
**5 µM**	**4509**	**-23**	**-149**	**-8**	**-67**
**10 µM**		**-12**	**-168**	**-7**	**-82**
**20 µM**		**+9**	**-167**	**-1**	**-90**
**5 µM**	**6167**	**-12**	**-66**	**-4**	**-29**
**10 µM**		**-3**	**-69**	**-2**	**-33**
**20 µM**		**+14**	**-61**	**+5**	**-31**
5 µM	9691	+3	-9	+5	-1
10 µM		+12	-4	+12	+5
20 µM		+29	+11	+25	+18

S2.2 Additional drug doses

In addition to the drug doses tested in the main manuscript, we performed an additional test using 100 µM ranolazine.

The following Table S10 summarizes the blocking effects of the two drugs in our simulations.

Table S10. Ionic current percent block for the drug doses tested for mexiletine and ranolazine.

	Mexiletine on Control population			Ranolazine on Control population			
	5 µM	10 µM	20 µM	5 µM	10 µM	20 µM	100 µM
I_{Na}	10.4 %	18.9 %	31.8 %	0.3 %	1.0 %	3.2 %	30.7 %
I_{NaL}	95.5 %	95.9 %	96.6 %	45.5 %	62.5 %	76.9 %	94.3 %
I_{CaL}	4.8 %	9.1 %	16.7 %	9.1 %	16.7 %	28.6 %	66.7 %
I_{Kr}	9.1 %	16.7 %	28.6 %	9.1 %	16.7 %	28.6 %	66.7 %
	Mexiletine on Mutant population			Ranolazine on Mutant population			
	5 µM	10 µM	20 µM	5 µM	10 µM	20 µM	100 µM
I_{Na}	27.3 %	42.9 %	60.1 %	2.8 %	5.9 %	11.9 %	44.8 %
I_{NaL}	65.8 %	79.4 %	88.5 %	33.7 %	45.7 %	58.3 %	81.9 %
I_{CaL}	4.8 %	9.1 %	16.7 %	9.1 %	16.7 %	28.6 %	66.7 %
I_{Kr}	9.1 %	16.7 %	28.6 %	9.1 %	16.7 %	28.6 %	66.7 %

Figure S10A shows how 100 µM ranolazine affects the control and mutant hiPSC-CM populations in the simulations. As for the other doses, the drugs affect differently the control and the mutant

populations. In particular, the number of control hiPSC-CM models producing spontaneous APs after drug administration are 1107 (692 showing an AP shortening and 415 showing AP prolongation). The number of mutant hiPSC-CM models producing APs after drug administration are 810 (605 showing AP shortening and 205 AP prolongation). On average, the APD_{90} shortening is -32.3 ms for the control population and -116.5 ms for the mutant one, as shown in Figure S10B. Finally, Figure S10C shows how 100 µM ranolazine affects the symptomatic and asymptomatic models: the global effect is APD shortening, and thus APD_{90} was shortened by 156 ms (-26.4%) for the symptomatic models and by 25 ms (-6.0%) for the asymptomatic ones. Interestingly, such dose of ranolazine restores symptomatic APD_{90} to the asymptomatic level on average.

As for 20 µM mexiletine, in the subgroup with positive ΔAPD_{90} in response to 100 µM ranolazine we noticed depolarized MDP (-4.7% median percent differences compared to the subgroup characterized by AP shortening) and reduced VMax (-76.1%), as well as a reduced I_{K1} (-31.8%) and I_{NaL} (-16.9%). In these conditions, a strong block of Na^+ currents as for ranolazine affects dramatically the AP upstroke, delaying it and increasing the APD.

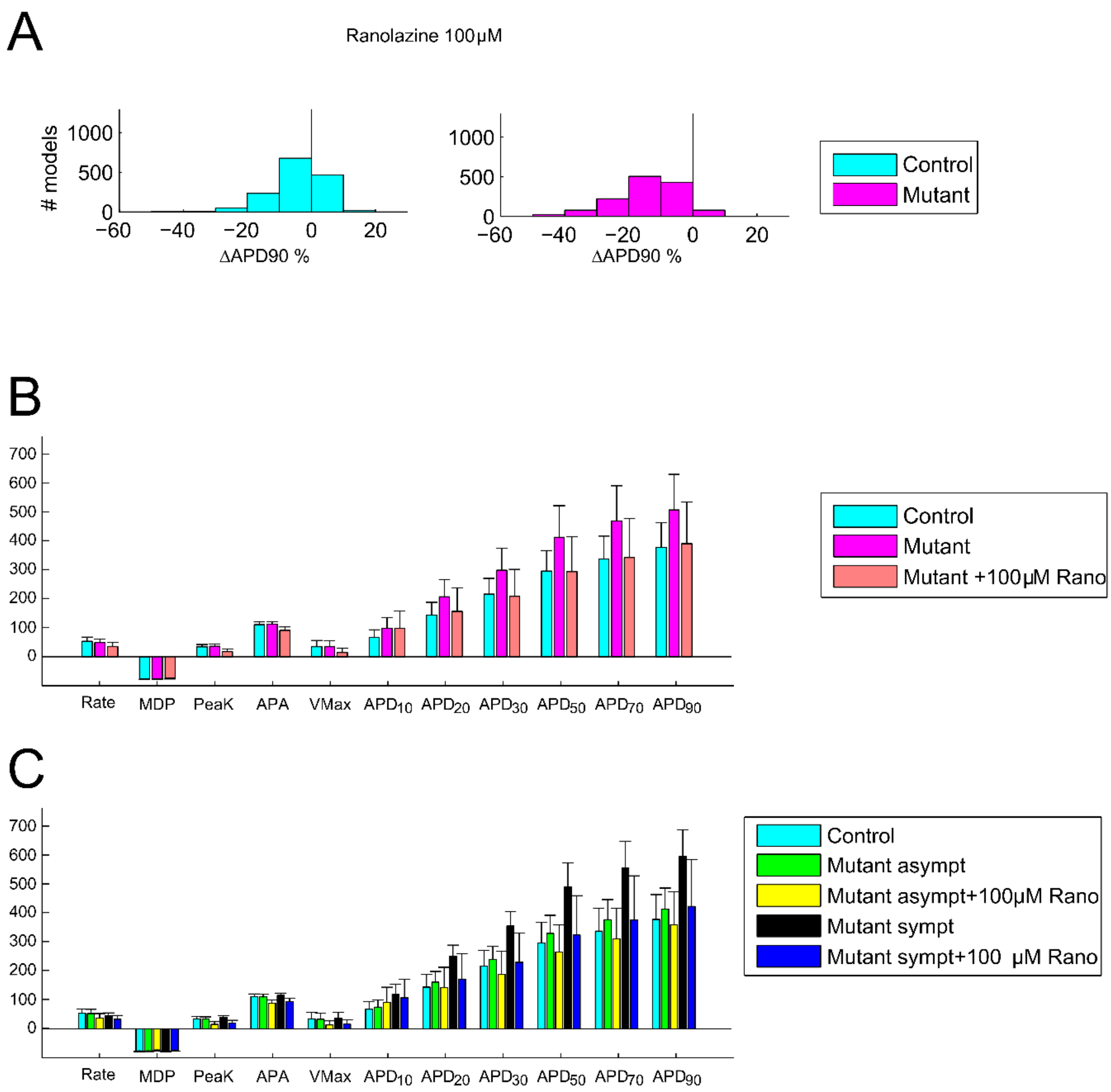

Figure S10. A) ΔAPD_{90} for the models of the control and LQT3 hiPSC-CM populations at 100 μM ranolazine. B) Ranolazine effects on AP biomarkers for the control and mutant hiPSC-CM populations. C) Ranolazine effects on AP biomarkers for the symptomatic and asymptomatic hiPSC-CM mutant models.

S1.3 Computing time and resources

Table S11. Computing time required for the population simulations on 32 cores, 8 GB memory/core on the Taito super cluster (www.csc.fi). All simulations were conducted in MATLAB (The Mathworks, Inc., Natick, MA, USA).

	Number of models	Simulation length (s)	Computing time (h)
CONTROL POPULATION	10000	800	48
MUTANT POPULATION	1463	800	3
DRUG TEST (e.g. 5 μM mexiletine)	1463	300	2

References

1. Paci M, Hyttinen J, Aalto-Setälä K, Severi S: Computational models of ventricular- and atrial-like human induced pluripotent stem cell derived cardiomyocytes. Ann Biomed Eng 2013; 41:2334–2348.

2. Paci M, Hyttinen J, Rodriguez B, Severi S: Human induced pluripotent stem cell-derived versus adult cardiomyocytes: an in silico electrophysiological study on effects of ionic current block. Br J Pharmacol 2015; 172:5147–5160.

3. O'Hara T, Virág L, Varró A, Rudy Y: Simulation of the Undiseased Human Cardiac Ventricular Action Potential: Model Formulation and Experimental Validation. PLoS Comput Biol 2011; 7:e1002061.

4. Ma D, Wei H, Zhao Y, et al.: Modeling type 3 long QT syndrome with cardiomyocytes derived from patient-specific induced pluripotent stem cells. Int J Cardiol 2013; 168:5277–5286.

5. Carbonell-Pascual B, Godoy E, Ferrer A, Romero L, Ferrero JM: Comparison between Hodgkin-Huxley and Markov formulations of cardiac ion channels. J Theor Biol Elsevier, 2016; 399:92–102.

6. Kennedy M, Bers D, Chiamvimonvat N, Sato D: Dynamical effects of calcium-sensitive potassium currents on voltage and calcium alternans. J Physiol 2017; In Press.

7. Britton OJ, Bueno-Orovio A, Van Ammel K, Lu HR, Towart R, Gallacher DJ, Rodriguez B: Experimentally calibrated population of models predicts and explains intersubject variability in cardiac cellular electrophysiology. Proc Natl Acad Sci U S A 2013; 110:E2098–E2105.

8. Ma J, Guo L, Fiene SJ, Anson BD, Thomson JA, Kamp TJ, Kolaja KL, Swanson BJ, January CT: High purity human-induced pluripotent stem cell-derived cardiomyocytes: electrophysiological properties of action potentials and ionic currents. AJP - Hear Circ Physiol 2011; 301:H2006–H2017.

9. Moretti A, Bellin M, Welling A, et al.: Patient-specific induced pluripotent stem-cell models for long-QT syndrome. N Engl J Med 2010; 363:1397–1409.

10. Fatima A, Kaifeng S, Dittmann S, Xu G, Gupta MK, Linke M, Zechner U, Nguemo F, Milting H, Farr M, Hescheler J, Šarić T: The disease-specific phenotype in cardiomyocytes derived from induced pluripotent stem cells of two long QT syndrome type 3 patients. PLoS One 2013; 8:e83005.

11. Lahti AL, Kujala VJ, Chapman H, et al.: Model for long QT syndrome type 2 using human iPS cells demonstrates arrhythmogenic characteristics in cell culture. Dis Model Mech. 2012, pp. 220–230.

12. Kujala K, Paavola J, Lahti A, et al.: Cell model of catecholaminergic polymorphic ventricular tachycardia reveals early and delayed afterdepolarizations. PLoS One 2012; 7:e44660.

13. Passini E, Mincholé A, Coppini R, Cerbai E, Rodriguez B, Severi S, Bueno-Orovio A:

Mechanisms of pro-arrhythmic abnormalities in ventricular repolarisation and anti-arrhythmic therapies in human hypertrophic cardiomyopathy. J Mol Cell Cardiol 2016; 96:72–81.

14. Duda RO, Hart PE, Stork DG: Pattern Classification. 2001,.

15. Wang DW, Yazawa K, Makita N, George AL, Bennett PB: Pharmacological targeting of long QT mutant sodium channels. J Clin Invest 1997; 99:1714–1720.

16. Moreno JD, Yang PC, Bankston JR, Grandi E, Bers DM, Kass RS, Clancy CE: Ranolazine for congenital and acquired late iNa-linked arrhythmias: In silico pharmacological screening. Circ Res 2013; 113.

17. Mirams GR, Cui Y, Sher A, Fink M, Cooper J, Heath BM, McMahon NC, Gavaghan DJ, Noble D: Simulation of multiple ion channel block provides improved early prediction of compounds' clinical torsadogenic risk. Cardiovasc Res 2011; 91:53–61.

18. Antzelevitch C, Belardinelli L, Zygmunt AC, Burashnikov A, Di Diego JM, Fish JM, Cordeiro JM, Thomas G: Electrophysiological effects of ranolazine, a novel antianginal agent with antiarrhythmic properties. Circulation 2004; 110:904–910.

19. Knollmann BC: Induced pluripotent stem cell-derived cardiomyocytes: boutique science or valuable arrhythmia model? Circ Res 2013; 112:969–976.
